# Efficacy of early PET-CT directed switch to carboplatin and paclitaxel based definitive chemoradiotherapy in patients with oesophageal cancer who have a poor early response to induction cisplatin and capecitabine in the UK: a multi-centre randomised controlled phase II trial

**DOI:** 10.1016/j.eclinm.2023.102059

**Published:** 2023-06-26

**Authors:** Somnath Mukherjee, Christopher N. Hurt, Richard Adams, Andrew Bateman, Kevin M. Bradley, Sarah Bridges, Stephen Falk, Gareth Griffiths, Sarah Gwynne, Christopher M. Jones, Philip J. Markham, Tim Maughan, Lisette S. Nixon, Ganesh Radhakrishna, Rajarshi Roy, Simon Schoenbuchner, Hamid Sheikh, Emiliano Spezi, Maria Hawkins, Thomas D.L. Crosby

**Affiliations:** a Oxford Cancer Centre, Oxford University Hospitals NHS Foundation Trust, Oxford, UK; b Centre for Trials Research, Cardiff University, Cardiff, UK; c Southampton Clinical Trials Unit, University of Southampton, Southampton, UK; d University Hospital Southampton NHS Foundation Trust, Southampton, UK; e Wales Research and Diagnostic Positron Emission Tomography Centre (PETIC), Cardiff University, Cardiff, UK; f University Hospitals Bristol NHS Foundation Trust, Bristol, UK; g South West Wales Cancer Centre, Swansea Bay University Health Board, Swansea, UK; h Department of Oncology, University of Cambridge, Cambridge, UK; i Oxford Institute for Radiation Oncology, University of Oxford, Oxford, UK; j The Christie Hospital, The Christie Hospitals NHS Foundation Trust, Manchester, UK; k Queen's Centre for Oncology, Hull University Teaching Hospitals NHS Trust, UK; l School of Engineering, Cardiff University, Cardiff, UK; m Department of Medical Physics & Biomedical Engineering, University College London, London, UK; n Velindre Cancer Centre, Velindre University NHS Trust, Cardiff, UK

**Keywords:** Oesophageal squamous cell carcinoma, Oesophageal adenocarcinoma, Chemoradiotherapy

## Abstract

**Background:**

The utility of early metabolic response assessment to guide selection of the systemic component of definitive chemoradiotherapy (dCRT) for oesophageal cancer is uncertain.

**Methods:**

In this multi-centre, randomised, open-label, phase II substudy of the radiotherapy dose-escalation SCOPE2 trial we evaluated the role of ^18^F-Fluorodeoxyglucose positron emission tomography (PET) at day 14 of cycle 1 of three-weekly induction cis/cap (cisplatin (60 mg/m^2^)/capecitabine (625 mg/m^2^ days 1–21)) in patients with oesophageal squamous cell carcinoma (OSCC) or adenocarcinoma (OAC). Non-responders, who had a less than 35% reduction in maximum standardised uptake value (SUV_max_) from pre-treatment baseline, were randomly assigned to continue cis/cap or switch to car/pac (carboplatin AUC 5/paclitaxel 175 mg/m^2^) for a further induction cycle, then concurrently with radiotherapy over 25 fractions. Responders continued cis/cap for the duration of treatment. All patients (including responders) were randomised to standard (50Gy) or high (60Gy) dose radiation as part of the main study. Primary endpoint for the substudy was treatment failure-free survival (TFFS) at week 24. The trial was registered with International Standard Randomized Controlled Trial Number 97125464 and ClinicalTrials.govNCT02741856.

**Findings:**

This substudy was closed on 1st August 2021 by the Independent Data Monitoring Committee on the grounds of futility and possible harm. To this point from 22nd November 2016, 103 patients from 16 UK centres had participated in the PET-CT substudy; 63 (61.2%; 52/83 OSCC, 11/20 OAC) of whom were non-responders. Of these, 31 were randomised to car/pac and 32 to remain on cis/cap. All patients were followed up until at least 24 weeks, at which point in OSCC both TFFS (25/27 (92.6%) vs 17/25 (68%); p = 0.028) and overall survival (42.5 vs. 20.4 months, adjusted HR 0.36; p = 0.018) favoured cis/cap over car/pac. There was a trend towards worse survival in OSCC + OAC cis/cap responders (33.6 months; 95%CI 23.1-nr) vs. non-responders (42.5 (95%CI 27.0-nr) months; HR = 1.43; 95%CI 0.67–3.08; p = 0.35).

**Interpretation:**

In OSCC, early metabolic response assessment is not prognostic for TFFS or overall survival and should not be used to personalise systemic therapy in patients receiving dCRT.

**Funding:**

10.13039/501100000289Cancer Research UK.

**Research in context**

**Evidence before this study**

Definitive chemoradiotherapy (dCRT) is an alternative to surgery in patients with oesophageal squamous cell carcinoma (OSCC) and an option for patients with oesophageal adenocarcinoma (OAC) who are unfit or unwilling for surgery. The systemic component of dCRT contributes to overall outcomes. These remain poor with systemic failure rates equalling 30–50%. Metabolic response assessment following the induction component of CRT has been shown to be predictive of overall outcomes and, in a single randomised controlled trial, it was used to enable effective individualisation of therapy. Early (day 14) assessment of metabolic response by ^18^F-Fluorodeoxyglucose positron emission tomography (PET) – computed tomography (CT) is also suggested to predict outcomes and allow for treatment personalisation in patients receiving neoadjuvant chemotherapy, albeit based on prospective but non-randomised trials. The potential value of an early PET-CT-directed switch in therapy in patients receiving the induction component of CRT is not known.

**Added value of this study**

This study demonstrates that early metabolic response, based on a reduction in maximum standardised uptake value (SUV_max_) of 35% or greater as determined by PET-CT, is not prognostic in OSCC, and is unlikely to be in OAC, in patients treated with dCRT. It also showed that a switch in induction chemotherapy from cisplatin and capecitabine to carboplatin and paclitaxel for metabolic non-responders results in inferior outcomes in OSCC and OAC.

**Implications of all the available evidence**

The use of early PET-CT to individualise systemic therapy based on metabolic response in patients receiving dCRT cannot be recommended in OAC or OSCC. Further studies are required to optimise the systemic component of dCRT, and to identify biomarkers to enable the personalisation and optimisation of induction therapy. It also remains unclear to what extent PET-CT directed treatment selection in dCRT is efficacious for patients with OSCC.

## Introduction

Oesophageal cancer is a leading global cause of morbidity and mortality, responsible for the loss of 9.8 million disability-adjusted life years and around 436,000 deaths each year.^1^ Two histopathological subtypes predominate; oesophageal adenocarcinoma (OAC) and oesophageal squamous cell carcinoma (OSCC); the latter of which represents ∼90% of all cases of oesophageal cancer worldwide despite higher rates of OAC in some Western countries e.g. United Kingdom (UK).^2^ For patients with locally advanced OSCC, definitive chemoradiotherapy (dCRT) forms a standard of care considered equivalent to surgical resection following neoadjuvant CRT (naCRT) or chemotherapy. In contrast, in cases of locally advanced OAC, dCRT is reserved for those unable/unwilling to undergo surgical resection.^3^

There is considerable evidence for the importance of a systemic component of dCRT. This was first established by the pivotal RTOG 85-01 study, in which the addition of concurrent chemotherapy significantly reduced rates of persistent local disease and improved overall survival.^4^ Several studies have subsequently confirmed the same but most patients still succumb to their disease and systemic failure rates of 30–50% persist.^5^, ^6^, ^7^, ^8^, ^9^ The SCOPE1 trial identified higher cisplatin dose intensity as an independent predictor of overall survival in patients treated with dCRT.^5^^,^^6^ Notably, however, concurrent chemotherapy was the major contributor to CRT-related toxicities in SCOPE1 as well as other dCRT trials.^6^^,^^10^, ^11^, ^12^

Given this, disease outcomes may be improved by optimising the systemic component of dCRT. Historically this comprised of a fluoropyrimidine and platinum doublet, though recent evidence suggests that equivalent outcomes may be achieved with lower toxicity through the use of carboplatin and paclitaxel.^4^^,^^11^, ^12^, ^13^, ^14^ There are no available biomarkers to guide selection of either chemotherapy doublet in the context of dCRT. In contrast, in the neoadjuvant setting, there has been considerable interest in the use of ^18^F-Fluorodeoxyglucose emission tomography (PET) – computed tomography (CT) for predicting response to chemotherapy.^15^, ^16^, ^17^, ^18^, ^19^ In the UK, two cycles of induction chemotherapy are routinely used prior to concurrent dCRT. We hypothesised that early PET-CT assessment of response to this induction treatment would guide selection of an optimal concurrent chemotherapy arm of dCRT in locally advanced OAC and OSCC.

This was assessed within the UK multi-centre phase II/III, open-label Study of Chemoradiotherapy in Oesophageal cancer including PET response and dose Escalation (SCOPE2) trial. This seeks to evaluate the impact of radiotherapy dose escalation but incorporates a second randomised phase II component focussed on early response assessment using PET-CT. In August 2021, the Independent Data Monitoring Committee (IDMC) recommended the PET-directed randomisation study component should stop on the grounds of futility and possible harm and allowed dissemination of the data. Herein we present the outcomes from this substudy of the ongoing SCOPE2 trial.

## Methods

### Study design and patients

The SCOPE2 randomised phase II/III, multicentre, open label, parallel, 2 × 2 factorial study was established to address two research questions. Firstly, whether high dose dCRT (60Gy in 25 fractions) improves overall survival compared to standard dose dCRT (50Gy in 25 fractions). Secondly, whether in non-responders to induction cis/cap (based on PET-CT imaging on day 14 of cycle 1), an early switch to car/pac reduces treatment failure at 24 weeks following the start of treatment (a predictor of overall survival).^5^

We recruited patients from radiotherapy centres in the UK with key eligibility criteria (full inclusion/exclusion criteria in Supplementary Table S1): histologically confirmed carcinoma of the oesophagus (OAC, OSCC or undifferentiated carcinoma) or gastro-oesophageal junction (Siewert Type 1 or 2 with less than 2 cm extension into the stomach), not including tumours with a proximal extent of less than 15 cm *ab oral*; selected for dCRT by a designated multi-disciplinary team; age 17 years or over; WHO performance status 0 or 1; T1-4 and node positive or negative (assessed by TNM 7); with a total disease length of 10 cm or less (amended to 13 cm or less from February 2019). M1 nodes encompassed within the radical radiotherapy volume were eligible.

Patients were required to have staging investigations including contrast-enhanced spiral CT scan of thorax, abdomen, pelvis and a PET-CT. Endoscopic ultrasound (EUS) was strongly recommended but not mandated. Eligible patients required adequate renal (EDTA glomerular filtration rate (GFR) greater than 40 ml/min, or estimated by Cockcroft-Gault formula >60 ml/min), liver (serum bilirubin≤1.5x upper limit of normal (ULN), ALT/AST ≤ 2.5x ULN, ALP ≤ 3x ULN), haematological (absolute neutrophil count (ANC) > 1.5 × 10^9^/l, platelet >100 × 10^9^/l), lung (FEV1>1.0) and cardiac function (echocardiogram/MUGA scan left ventricular ejection fraction (LVEF) > 40%) if of clinical concern. Patients had to provide written informed consent prior to registration.

Participation in the PET response substudy described in this paper was optional for patients and treating centres and required additional eligibility assessments: commenced first induction cycle with cis/cap, baseline PET scan <5 weeks prior to start of induction chemotherapy with SUV_max_ of 5 or more, PET-CT repeated on day 14 (−2/+3 days) of their first induction cycle. In keeping with neoadjuvant studies, undertaken in the context of neoadjuvant chemotherapy, non-responders were defined as those who showed <35% reduction in SUV_max_ and ≥35% defined responders.^13^^,^^14^^,^^17^ See SCOPE2 PET Imaging Manual in Supplementary Materials.

The protocol for SCOPE2 has been published elsewhere.^20^ The study was approved by the UK Medicines and Healthcare products Regulatory Agency and the Wales Research Ethics Committee (15/WA/0395). Velindre University NHS Trust is sponsor and the study is coordinated by the Centre for Trials Research (CTR) at Cardiff University. A Trial Management Group and independent Trial Steering Committee oversaw the study, with an IDMC reviewing recruitment, toxicity, compliance and survival data at six-monthly intervals.

### Randomisation and masking

Randomisation was conducted once 14 day PET response was known, prior to starting cycle 2 of induction treatment. All patients were randomised 1:1 to standard or high dose radiotherapy (Supplementary Figure S1). Non-responders were additionally randomised (1:1) to continue cis/cap or switch to car/pac chemotherapy which forms the basis of this paper. The randomisation was stratified by recruiting hospital, primary reason for not having surgery, and stage using the method of stratified minimisation with a random element (80:20) and was conducted separately in the OAC and OSCC cohorts. Concealed allocation was achieved by nurses (who recruited the patients) telephoning the CTR, where randomisation was performed by a Trial/Data Manager interacting with a bespoke computerised system.

### Procedures

#### Treatments

All patients included in this analysis had the same first cycle of induction cis/cap chemotherapy, comprising of intravenous cisplatin (60 mg/m^2^) on day one and oral capecitabine (625 mg/m^2^) twice daily for three weeks. Patients categorised as responders on PET-CT continued this treatment for three further cycles, with cycles 3 and 4 given concomitantly with external beam radiotherapy (EBRT) at standard dose (50Gy in 25 fractions) or at a high dose (60Gy in 25 fractions).

For non-responders, patients randomised to the car/pac arm received a three week cycle of intravenous carboplatin (AUC 5) and intravenous paclitaxel (175 mg/m^2^), both delivered on day 1; followed by weekly intravenous carboplatin (AUC 2) and intravenous paclitaxel (50 mg/m^2^) given concomitantly with standard-dose (50Gy in 25 fractions) or high dose (60Gy in 25 fractions) EBRT. Patients allocated to the cis/cap treatment received 3 further cycles of three weekly intravenous cisplatin (60 mg/m^2^) on day one and oral capecitabine (625 mg/m^2^ twice daily) on days 1–21 with last two cycles given concomitantly with standard-dose (50Gy in 25 fractions) or high dose (60Gy in 25 fractions) EBRT.

If patients were unable to swallow capecitabine, investigators were permitted to instead use intravenous 5-fluorouracil (5-FU; 225 mg/m^2^/day) infusion extending from day 1 through 21 of each cycle. Where intravenous cisplatin was considered contraindicated, intravenous carboplatin (AUC 5) was permitted. Contraindications to cisplatin included, but were not restricted to, advanced age, poor renal function, concerns regarding neurotoxicity or clinically significant hearing impairment. Chemotherapy dose modifications are summarised in the Supplementary Materials.

An intravenous contrast-enhanced 3D-CT or 4D-CT in treatment position with slice thickness of no greater than 3 mm was acquired and a single-phase inverse-planned intensity modulated radiation therapy (IMRT) plan was produced. The gross tumour volume (GTV) is defined using all available imaging modalities. The target volumes were created as detailed in a comprehensive SCOPE2 radiotherapy planning guidance document, with protocols for middle third, upper third and 4D-CT cases included. External beam radiotherapy, prescribed in accordance with ICRU 50/62, was then delivered using multiple fields, Rapid Arc or volumetric modulated arc therapy (VMAT) techniques to a dose of 50Gy (standard dose) or 60Gy (high dose) in 25 fractions given five days per week. Details of the radiotherapy quality assurance process are contained within the Supplementary Materials.

#### Assessments

Patients were reviewed within three days prior to the first day of each cycle of induction chemotherapy and weekly whilst receiving concurrent CRT. Assessment at each review consisted of a medical examination and recording of toxicity evaluation as per Common Terminology Criteria of Adverse Events (NCI CTCAE version 4.03). An additional assessment was conducted at the end of treatment in week 12. Blood tests including full blood count and biochemical profile were done at each assessment.

Subsequent follow-up visits (including toxicity assessment) were conducted in weeks 15 and 24, and then 9, 12, 16, 20, 24, 36, 48, 60 months after enrolment. All patients underwent CT surveillance (and, where possible, endoscopic assessment) at week 24 ± 4 weeks (in response to the coronavirus pandemic in 2020, endoscopies performed as late as week 36 were accepted). No further imaging was mandated and subsequent investigations were based on patient symptomatology. The choice of second line treatment, including salvage surgery in the case of locoregional recurrence, was left to the discretion of the treating clinician.

#### Endpoints

The primary endpoint of the substudy was treatment failure-free survival (TFFS) at week 24 in PET non-responders. This was defined as patients being alive with no evidence of disease progression assessed by CT imaging and, wherever possible, with endoscopy and biopsy. Secondary endpoints were: acute toxicity (defined as that occurring within 12 weeks of the end of treatment^21^), overall survival, and progression-free survival (PFS).

### Statistical analysis

This trial was powered separately for OSCC and OAC (including undifferentiated) cohorts. At the time of designing the study, the 24 week TFFS in PET non-responders was unknown but was conservatively estimated to be 55% for OSCC. To detect an improvement to 75% with car/pac (chi square, 87% power, 0.2 one sided alpha), allowing for 7% loss to follow-up, would require 86 OSCC patients. We predicted that only 27 OAC patients would be recruited, which would be sufficient to detect an improvement in 24 week TFFS from 55% with cis/cap to 85% following a switch to car/pac (chi square,80% power, 0.20 one sided alpha), allowing for 5% loss to follow up.

Statistical analyses were performed using Stata version 17 according to intention to treat unless otherwise specified. All analyses were conducted according to a pre-specified analysis plan except where indicated as *post hoc*. In the OSCC cohort, the primary endpoint was analysed using chi square and mixed effects logistic regression that included the randomisation stratification criteria (with treating centre as a random effect). Post hoc sensitivity analyses also included some potentially prognostic baseline and treatment variables that looked imbalanced. In the OAC cohort, the failure-free rate was compared between arms using chi square and was not adjusted for multiple testing as these analyses relied on small numbers and were exploratory. The distribution of baseline SUV_max_ and percentage change in SUV_max_ between baseline and day 14 was compared between treatment failure and failure-free patients at 24 weeks in each trial arm separately using Wilcoxon rank sum tests. The proportions of patients with any grade 3 or 4 acute toxicity were compared between trial arms using a chi square test in all tumour types together in patients receiving at least one dose of randomised trial treatment. For survival analyses, we calculated survival time from date of randomisation to when an event occurred; whether this be progression or any death for PFS, and any death for overall survival. Patients who were event free were censored at the time they were last known to be event free. We estimated event time distributions with the Kaplan–Meier method. Cox regression was used to generate hazard ratios in:

i. a univariable model including trial arm in the OSCC cohort.
ii. univariable and multivariable models including trial arm, age, sex, WHO performance status, disease stage, tumour histology, and disease length in the combined OSCC + OAC cohort

In order to assess the prognostic value of 35% reduction in SUV_max_, we compared TFFS (using chi-square in each tumour type separately) and overall survival (using univariable cox-regression in the combined tumour types) between responders (who all received cis/cap) and non-responders randomised to cis/cap.

This trial is registered with International Standard Randomized Controlled Trial Number 97125464 and ClinicalTrials.gov NCT02741856.

### Role of the funding source

The funder of the study had no role in study design, data collection, data analysis, data interpretation, the writing of the study report or the decision to submit for publication. CH, SS, and PM had full access to the study data. All authors had final responsibility for the decision to submit for publication.

## Results

### Overview

**Fig. 1:**
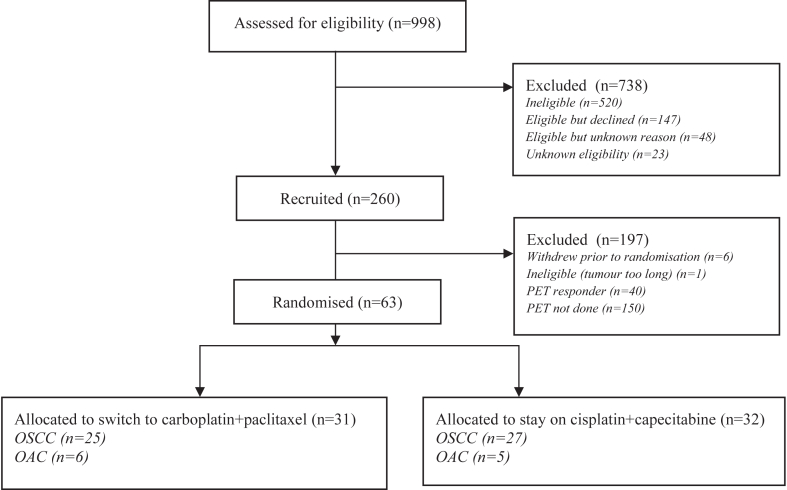
**Flow diagram of trial participants**.

The first patient was recruited on 22nd November 2016 and the IDMC recommended that the PET-CT-based substudy should be closed on 1st August 2021. At this point, 103 patients had been recruited from 16 centres (Supplementary Table S2) in the PET-CT substudy (Fig. 1) of whom 40 were responders and 63 non-responders (52 OSCC, 11 OAC) of whom 31 were allocated to switch to car/pac, whilst 32 were allocated to remain on treatment with cis/cap.

### Decision to stop randomisation in the substudy

When the PET-CT substudy closed, 14 of 20 (70.0%) OSCC patients randomised to car/pac were treatment failure-free at 24 weeks compared with 22 of 24 (91.7%) OSCC patients who continued with cis/cap (Supplementary Table S3). This data gave a 4% chance (conditional power) of seeing a significant result at the end of the study, with a similar pattern seen for OAC (Supplementary Table S3). The IDMC recommended: (i) stopping the chemotherapy switch randomisation on the basis of futility and potential harm and, (ii) continuing follow up of those patients to 24 weeks before conducting the analysis presented below.

### Continued cis/cap versus a switch to car/pac in non-responders

The baseline characteristics of the non-responders randomised to either continue with cis/cap or to switch to car/pac are shown in Table 1 (OSCC) and Table 2 (OAC). WHO performance status and gender do not appear well balanced between trial arms in the OSCC cohort. Median follow up in those still alive was 17.7 months (range: 5.4–46.7) in car/pac arm and 23.7 (range: 8.6–48.3) in cis/cap.

**Table 1:** Baseline patient characteristics–squamous cell carcinoma cohort.

	carboplatin + paclitaxel		cisplatin + capecitabine	
**Number randomised**	25		27	
**Median age (years), IQR**	67.7, 59.2–70.8		69.6, 61.9–72.2	
**Sex**				
Male	9	(36.0)	15	(55.6)
Female	16	(64.0)	12	(44.4)
**WHO performance status**				
0	8	(32.0)	16	(59.3)
1	17	(68.0)	11	(40.7)
**Reason for non-surgical therapy**				
Clinician's choice	16	(64.0)	16	(59.3)
Co-morbidity/Poor performance status	3	(12.0)	1	(3.7)
Local extent of disease	3	(12.0)	4	(14.8)
Patient choice	3	(12.0)	6	(22.2)
**T - Stage**				
T2	1	(4.0)	3	(11.1)
T3	19	(76.0)	18	(66.7)
T4a	1	(4.0)	4	(14.8)
T4b	4	(16.0)	2	(7.4)
**N - Stage**				
N0	10	(40.0)	12	(44.4)
N1	13	(52.0)	13	(48.2)
N2	2	(8.0)	2	(7.4)
**Histologic grade**				
G1	1	(4.0)	0	(0.0)
G2	19	(76.0)	17	(63.0)
G3	5	(20.0)	10	(37.0)
**Screening TNM v7 Stage**				
IIa	2	(8.0)	3	(11.1)
IIb	7	(28.0)	8	(29.6)
III	16	(64.0)	16	(59.3)
**Site of predominant tumour**				
Upper 1/3 (14 to <24 cm)	6	(24.0)	4	(14.8)
Mid-point (24 to <32 cm)	12	(48.0)	16	(59.3)
Lower 1/3 (32–40 cm)	7	(28.0)	7	(25.9)
**Median overall length of primary tumour (cm)**, IQR	5.1, 3.9–5.9		4.2, 3.0–5.4	
**Total disease length (cm)**, IQR	6.0, 4.6–8.0		5.0, 3.0–7.0	
**Baseline SUV**_**max**_**– median IQR**	13.6 (12.3–15.5)		13.4 (11.0–15.8)	
**% change in SUV**_**max**_**to day 14 - median IQR**^a^	18.9 (−1.7 to 25.3)		16.4 (9.0–23.7)	

NB. n (%) unless otherwise specified.

a 100 x (SUV_max_ at 14 days/SUV_max_ at baseline).

**Table 2:** Baseline patient characteristics–adenocarcinoma/undifferentiated histology cohort.

	carboplatin + paclitaxel		cisplatin + capecitabine	
**Number randomised**	6		5	
**Median age (years), IQR**	73.8, 70.1–76.3		71.4, 60.3–72.0	
**Gender**				
Male	5	(83.3)	4	(80.0)
Female	1	(16.7)	1	(20.0)
**WHO performance status**				
0	4	(66.7)	3	(60.0)
1	2	(33.3)	2	(40.0)
**Reason for non-surgical therapy**				
Clinician's choice	1	(16.7)	1	(20.0)
Co-morbidity/Poor performance status	2	(33.3)	1	(20.0)
Local extent of disease	2	(33.3)	1	(20.0)
Patient choice	1	(16.7)	2	(40.0)
**T - Stage**				
T1	0	(0.0)	1	(20.0)
T2	1	(16.7)	0	(0.0)
T3	4	(66.7)	3	(60.0)
T4a	1	(16.7)	1	(20.0)
**N - stage**				
N0	1	(16.7)	3	(60.0)
N1	4	(66.7)	0	(0.0)
N2	1	(16.7)	2	(40.0)
**Histologic grade**				
G1	1	(16.7)	0	(0.0)
G2	4	(66.7)	3	(60.0)
G3	1	(16.7)	1	(20.0)
**Screening TNM v7 Stage**				
I	0	(0.0)	1	(20.0)
IIb	2	(33.3)	2	(40.0)
III	4	(66.7)	2	(40.0)
**Site of predominant tumour**				
Mid-point (24 to <32 cm)	2	(33.3)	1	(20.0)
Lower 1/3 (32–40 cm)	4	(66.7)	4	(80.0)
**Median overall length of primary tumour (cm)**, IQR	4.9, 3.0–6.0		4, 3.7–7.0	
**Total disease length (cm)**, IQR	5.7, 4.0–7.4		4.5, 4.0–5.0	
**Baseline SUV**_**max**_**– median IQR**	8.9 (7.5–10.6)		8.5 (7.6–8.7)	
**% change in SUV**_**max**_**to day 14 - median IQR**^a^	7.9 (−8.0-25.5)		7.1 (−5.3-20.3)	

NB. n (%) unless otherwise specified.

a 100 × (SUV_max_ at 14 days/SUV_max_ at baseline).

**Fig. 2:**
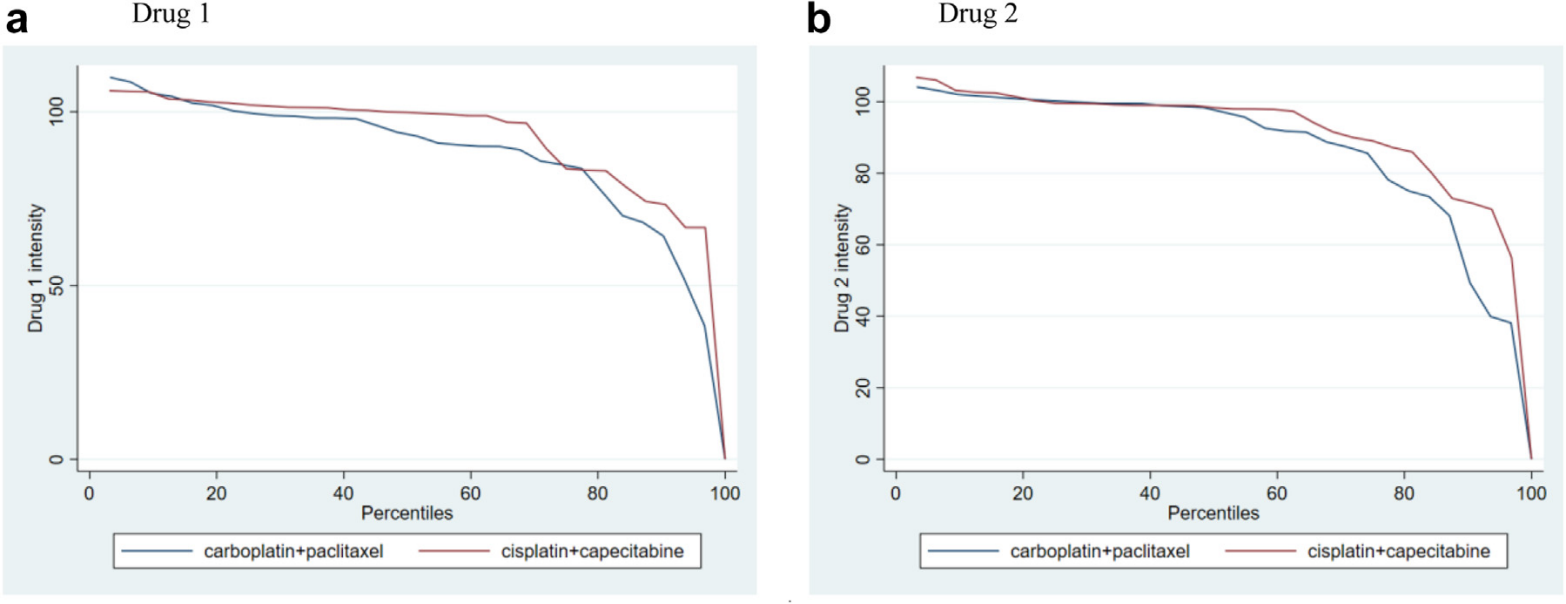
**Chemotherapy dose intensity (as % of full protocol dose) for each drug during cycles 2–4 by trial arm**. NB “drug 1” is carboplatin or cisplatin and “drug 2” is paclitaxel or capecitabine.

A higher proportion of patients were randomised to receive higher-dose radiotherapy in the cis/cap arm (n = 18; 56.3%) than in the car/pac arm (n = 14; 45.2%). Overall, more than 80% of patients received radiotherapy as per protocol in both trial arms (Table 3). Compliance with chemotherapy protocol treatment is shown in Table 4 and Fig. 2. More than 90% of patients received cycle 1 at full protocol dose in both trial arms. Dose intensity remained high in both treatment arms during cycles 2 to 4, with 92.9% (IQR 83.5–99.5) and 97.1% (IQR 78.2–100.2) respectively receiving per protocol dose platinum and paclitaxel, and 99.7% (83.4–101.8) and 98.2% (88.1–99.6) respectively receiving per protocol dose platinum and fluoropyrimidine. One patient with OAC in the cis/cap arm received no treatment after cycle 1 because they were found to have progressed soon after randomisation.

**Table 3:** Radiotherapy treatment.

Patients randomised	carboplatin + paclitaxel		cisplatin + capecitabine	
	31		32	
	n	%	n	%
Planned dose				
50Gy	17	54.8	14	43.8
60Gy	14	45.2	18	56.3
Planned 50Gy∗				
Given 50Gy in 25#	15	88.2	12	85.7
Other dose given	2^a^	11.8	1^b^	7.1
No RT given	0	0.0	1	7.1∗∗
Planned 60Gy∗				
Given 60Gy in 25#	10	71.4	16	88.9
Other dose given	4^c^	28.6	2^d^	11.1
No RT given	0	0.0	0	0.0
Days from randomisation to start of RT – median (IQR)	26 (25–32)		26 (24–27)	
Delays during RT				
Yes	3	9.7	5	15.6
No	28	90.3	26	81.3
N/A – no RT given	0	0.0	1	3.1
Median delay in days (IQR)	8 (1–33)		4 (1–7)	
Reasons for delay				
Toxicity	0	0.0	3	9.4
Patient choice	1	3.2	0	0.0
Stent/replan/other treatment	2	6.5	1	3.1
Bank holiday	0	0.0	1	3.1

Denominator for % is randomized patients except for ∗ where denominator is number of patients with this planned dose ∗∗withdrew from treatment after cycle 1 due to disease progression.

a 48Gy in 24# due to toxicity; 50Gy in 5# and 40gy in 20#.

b 36Gy in 18#.

c 50Gy in 25#; 51Gy in 27#; 50Gy in 25#; 60Gy in 5# and 48Gy in 20#.

d 24Gy in 12#; 60Gy in 1# and 57.6Gy in 3# and 42Gy in 21#.

**Table 4:** Chemotherapy treatment.

	carboplatin^a^ + paclitaxel^b^			cisplatin^a^ + capecitabine^b^		
Randomised		**31**			**32**	
**Cycle 1**						
Cisplatin						
	Replaced with carboplatin	5	16.1	Replaced with carboplatin	1	3.1
	Full dose	28	90.3	Full dose	29	90.6
	Reduced dose	3	9.7	Reduced dose	3	9.4
	No drug given	0	0.0	No drug given	0	0.0
Capecitabine	Replaced with 5FU	2	6.5	Replaced with 5FU	2	6.3
	Full dose	28	90.3	Full dose	30	93.8
	Reduced dose	3	9.7	Reduced dose	2	6.3
	No drug given	0	0.0	No drug given	0	0.0
**Cycle 2–4**						
Drug 1				Replaced with carboplatin for any cycle	2	6.3
	No drug given	1	3.2	No drug given	1	3.1
	Dose intensity % (IQR, R)	92.9 (83.7–99.5, 0–109.9)		Dose intensity % (IQR, R)	99.7 (83.4–101.8, 0–106.1)	
Drug 2				Replaced with 5FU for any week	2	6.3
	No drug given	1	3.2	No drug given	1	3.1
	Dose intensity % (IQR, R)	97.1 (78.2–100.2, 0–104.2)		Dose intensity % (IQR, R)	98.2 (88.1–99.6, 0–106.8)	
	Time from start of cycle 2 to start of cycle 4 (days – median (IQR), n)^c^	42 (41–42), 27		Time from start of cycle 2 to start of cycle 4 (days – median (IQR), n)^c^	42 (41–47), 31	

a Drug 1.

b Drug 2.

c Only those that received cycle 4 (week 10) of treatment.

All patients were assessable for TFFS (Table 5). In the OSCC cohort, the failure-free rate was 17/25 (68.0%) in the car/pac arm, compared with 25/27 (92.6%) in the cis/cap arm (λ^2^ = 5.054, p = 0.025; adjusted logistic regression p = 0.028; also adjusted for planned RT dose post-hoc p = 0.032; and sex and WHO PS post-hoc p = 0.039). The direction of failure rate trend favouring cis/cap was consistent across subgroups. The results in the OAC cohort are also given but the sample size was small (n = 11). Neither baseline SUV_max_ nor percentage change in SUV_max_ between baseline and day 14 were found to be associated with failure-free rate at 24 weeks in either treatment arm (Supplementary Figure S2).

**Table 5:** 24 Week treatment failure-free survival (TFFS) – n (%).

	carboplatin + paclitaxel		cisplatin + capecitabine		Odds Ratio (95% CI)	p value
**Squamous cell**						
**Patients randomised**	25		27			
Died/progressed prior to week 24 scan^a^	3	12.0	0	0.0		
Valid CT scan done^b^	22	88.0	27	100.0		
Progression outside RT volume	4	16.0	2	7.4		
Valid endoscopy done^c^	21	84.0	23	85.2		
Residual/persistent disease	3	12.0	0	0.0		
Response assessable	25	100.0	27	100.0		
Failure-free	17	68.0	25	92.6	0.14 (0.02–0.81)	0.028^d^
Failure	8	32.0	2	7.4		
**Adenocarcinoma and undifferentiated**						
**Patients randomised**	6		5			
Died/progressed prior to week 24 scan^a^	1	16.7	1	20.0		
Valid CT scan done^b^	5	83.3	4	80.0		
Progression outside RT volume	0	0.0	0	0.0		
Valid endoscopy done^c^	5	83.3	3	60.0		
Residual/persistent disease	1	16.7	0	0.0		
Response assessable	6	100.0	5	100.0		
Failure-free	4	66.7	4	80.0	0.50 (0.03–9.46)	0.621^e^
Failure	2	33.3	1	20.0		
**Subgroup analyses of failure-free rate**						
Age						
<70	10	62.5	15	93.8		
≥70	11	73.3	14	87.5		
Sex						
Male	9	64.3	18	94.7		
Female	12	70.6	11	84.6		
WHO status						
0	8	66.7	17	89.5		
1	13	68.4	12	92.3		
Stage						
I or II	8	72.7	14	100.0		
III	13	65.0	15	83.3		
Total disease length						
<5 cm	8	80.0	13	92.9		
≥5 cm	13	61.9	16	88.9		

a Therefore no CT scan due at 24 weeks.

b Done at 24 weeks ( ± 4 weeks) after start of treatment.

c Done at 24 weeks (+12/-4 weeks) after start of treatment.

d Mixed effects logistic regression.

e Chi square.

A summary of toxicities of all grades from the start of treatment in cycle 2 to week 24 is provided in Supplementary Table S4. Grade 3/4 toxicities are summarised in Table 6. Overall rates are very similar between the arms, with 22/31 (71.0%) treated with car/pac experiencing a grade 3/4 toxicity compared with 21/31 (67.7%) treated with cis/cap (λ^2^ = 0.0759, p = 0.783). The most common toxicity at grade 3/4 was dysphagia, experienced by 14/31 (45.2%) of those treated with car/pac, and 11/31 (35.5%) of those treated with cis/cap.

**Table 6:** CTCAE Grade 3/4 toxicity up to week 24.

	carboplatin + paclitaxel		cisplatin + capecitabine	
	n	%	n	%
**Randomised**	31		32	
**Received at least one dose of assigned chemotherapy**^a^	31		31	
**Any Grade 3 or 4**	22	71.0	21	67.7
**Blood and lymphatic system disorders**				
Febrile neutropenia	3	9.7	1	3.2
Anaemia	2	6.5	1	3.2
**Cardiac disorders**				
Cardiac arrest	1	3.2	0	0.0
Conduction disorder/atrial fibrillation	1	3.2	1	3.2
Heart failure	0	0.0	1	3.2
**Ear and labyrinth disorders**				
Hearing impaired	1	3.2	0	0.0
**Gastrointestinal disorders**				
Abdominal pain	0	0.0	1	3.2
Colon perforation	0	0.0	1	3.2
Diarrhoea	1	3.2	1	3.2
Dysphagia	14	45.2	11	35.5
Nausea/vomiting	1	3.2	3	9.7
Oesophageal pain	0	0.0	1	3.2
Oesophageal stenosis	0	0.0	1	3.2
Oesophagitis	3	9.7	7	22.6
**General disorders and administration site conditions**				
Fatigue	2	6.5	1	3.2
Fever	0	0.0	1	3.2
**Infections and infestations**				
Lung infection	1	3.2	1	3.2
Sepsis	0	0.0	1	3.2
Skin infection	2	6.5	0	0.0
Other infections	1	3.2	2	6.5
**Investigations**				
Lymphocyte decrease	1	3.2	1	3.2
Neutrophil decrease	5	16.1	5	16.1
Platelet decrease	1	3.2	2	6.5
Weight loss	0	0.0	1	3.2
**Metabolism and nutrition disorders**				
Anorexia	1	3.2	3	9.7
Dehydration	2	6.5	2	6.5
Hypokalaemia	1	3.2	2	6.5
Hypomagnesemia	0	0.0	1	3.2
**Nervous system disorders**				
Stroke	0	0.0	2	6.5
Syncope	1	3.2	0	0.0
**Psychiatric disorders**				
Acute confusion	1	3.2	0	0.0
**Respiratory, thoracic and mediastinal disorders**				
Aspiration	0	0.0	2	6.5
Dyspnoea	1	3.2	1	3.2
Pneumonitis	1	3.2	0	0.0
**Skin and subcutaneous tissue disorders**				
Alopecia	1	3.2	0	0.0
**Vascular disorders**				
Hypotension	1	3.2	1	3.2
Thromboembolic event	0	0.0	2	6.5

a This forms the denominator in this table.

**Fig. 3:**
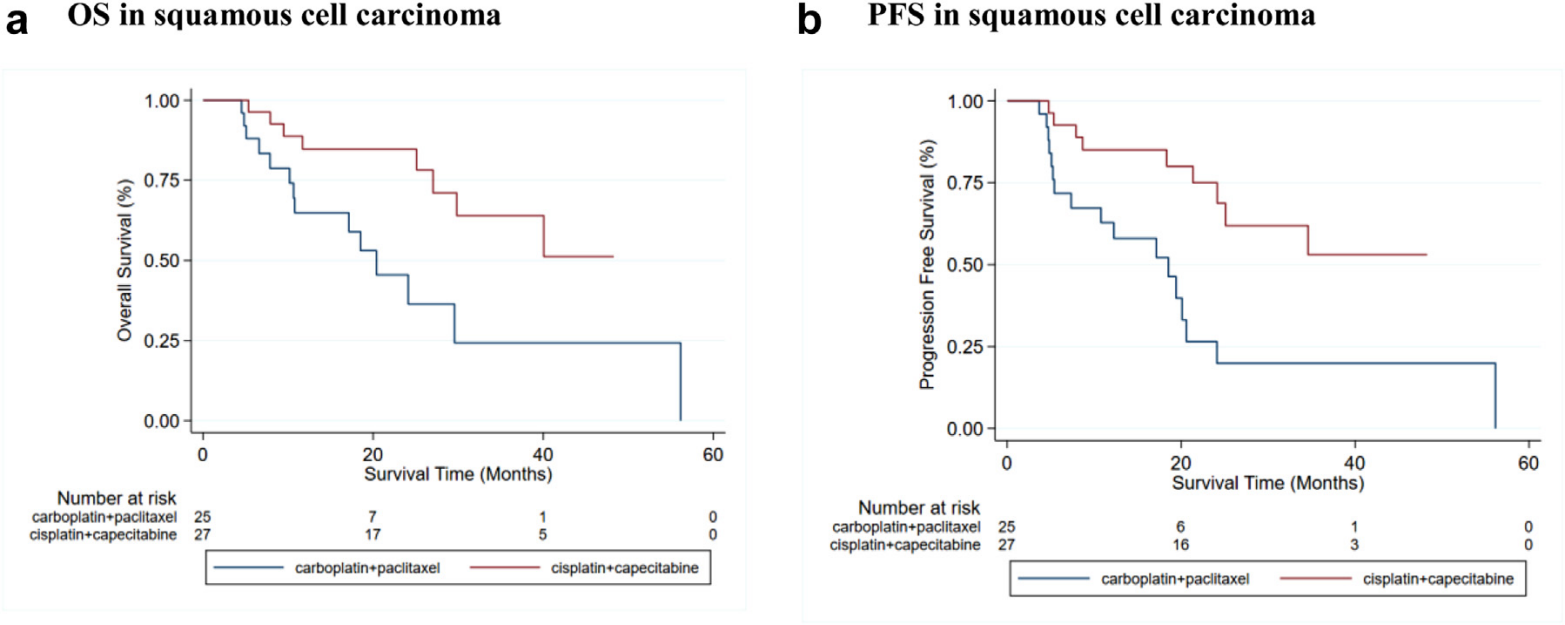
**Kaplan Meier curves of overall (OS) and progression free (PFS) survival**.

For patients with OSCC, overall survival was significantly better in the cis/cap arm (Fig. 3a, unadjusted HR = 0.32 [95%CI: 0.13–0.79], p = 0.013, n = 52). In OSCC + OAC cohorts combined (Supplementary Table S5), overall survival was significantly better in the cis/cap arm (median 20.4 [95%CI 10.8–43.4] versus 42.5 [95%CI 27.0-not reached (nr)] months; unadjusted HR = 0.44 [95%CI: 0.20–0.97, p = 0.041; adjusted HR = 0.36 [95%CI: 0.16–0.84], p = 0.018, n = 63). A better WHO performance status, but nothing else, was also significantly associated with improved overall survival (unadjusted HR = 2.41 [95%CI: 1.06–5.48, p = 0.036; adjusted HR = 2.96 [95%CI: 1.19–7.38], p = 0.036, n = 63). Causes of death are shown in Supplementary Table S6.

For patients with OSCC, PFS was significantly better in the cis/cap arm (Fig. 3b, unadjusted HR = 0.30 [95%CI: 0.13–0.69], p = 0.005, n = 52). In OSCC + OAC cohorts combined (Supplementary Table S7), PFS was higher in the cis/cap arm although this was not statistically significant (median 19.4 [95%CI: 10.8–24.1] versus 34.6 [95%CI: 18.3-nr] months; unadjusted HR = 0.54 [95%CI: 0.27–1.08, p = 0.079; adjusted HR = 0.58 [95%CI: 0.28–1.21], p = 0.147, n = 63). Further, if the patient who progressed prior to cycle 2 is removed from the analysis of all patients, then the evidence for cis/cap having better PFS strengthens (adjusted HR = 0.42 [95%CI: 0.20–0.91], post hoc p = 0.027, n = 62). No other variables were significantly associated with PFS.

Patterns of first progression by trial arm and histopathological subtype are summarised in Supplementary Table S8. In the OSCC cohort, 8/17 (47.1%) of first progression events were deaths in the car/pac arm compared to 3/9 (33.3%) in the cis/cap arm. In the OAC cohort, 3/3 (100%) first progression events were deaths in the car/pac arm compared to 0/5 (0%) in the cis/cap arm. Overall, 10/34 (29.4%) involved distant progressions.

### Responder vs non-responder outcomes

Forty (38.8%) of the 103 patients recruited to the PET-CT substudy were responders. In comparing outcomes between responders (who all received cis/cap, n = 40) and non-responders (n = 63), we have considered only non-responding patients who received cis/cap (n = 32) to assess the prognostic value of 35% reduction in SUV_max_. Baseline characteristics (Supplementary Table S9) are similar in each group, though baseline SUV_max_ was higher in the responders (post hoc t test = −3.5614, p < 0.001). In the OSCC cohort, the failure-free rate was 25/27 (92.6%) for non-responders and 22/28 (78.6%) for responders (λ^2^ = 2.1740, p = 0.140) and, similarly, in the OAC cohort the failure-free rate was 4/5 (80.0%) in non-responders and 7/9 (77.8%) in responders (λ^2^ = 0.0094, p = 0.923) (Supplementary Table S10). There was a similar direction of trend in median overall survival (Supplementary Figure S3), which measured 42.5 (95% CI: 27.0-nr) months in non-responders receiving cis/cap and 33.6 (23.1-nr) months in responders (univariable HR = 1.43, 95CI: 0.67–3.08, p = 0.350, n = 72) for patients with OAC and OSCC combined. In this same group, there was no significant difference in the distribution of baseline SUV_max_ between those who failed at 24 weeks and those who did not (post hoc z = −1.166, p = 0.2489).

## Discussion

The phase II PET-CT substudy of the SCOPE2 trial was stopped by the IDMC on the grounds of futility and possible harm. In OSCC, switching from cis/cap to car/pac chemotherapy based on <35% reduction in SUV_max_ on PET-CT at day 14 of cycle 1 of induction treatment significantly worsens 24 week TFFS and overall survival. The sample size was too small in the OAC cohort to draw meaningful conclusions. Treatment compliance was high in both trial arms with similar rates of grade 3/4 toxicity. Although not significant, there were trends towards better 24 week TFFS and overall survival in non-responders randomised to cis/cap when compared to responders (who all got cis/cap). This suggests that a 35% reduction in SUV_max_ during induction chemotherapy is not prognostic of better outcome in patients managed with dCRT.

This study was undertaken in the context of a malignancy for which outcomes following CRT remain poor, with a median overall survival of 2–3 years in contemporary trials.^5^^,^^6^^,^^22^^,^^23^ Previous attempts to improve outcomes using radiotherapy dose escalation and the inclusion of targeted therapies have failed and there are a paucity of available biomarkers to guide either the systemic or radiotherapy components of dCRT.^5^^,^^6^^,^^22^, ^23^, ^24^, ^25^ The importance of intensified systemic treatment is, however, increasingly clear and, as such, there has been growing interest in means to optimise and personalise this aspect of dCRT.

Although not advocated for within European guidance, induction chemotherapy is a standard feature of dCRT within the UK.^3^^,^^6^ There is a lack of randomised evidence to guide the use of induction chemotherapy in OAC or OSCC, and there is considerable discord within the literature with respect to induction chemotherapy use for CRT in the context of SCCs of the head, neck and anus.^26^, ^27^, ^28^ Whilst there are no randomised, controlled data evaluating its use in this context, the theoretical advantages include an early improvement in dysphagia and elimination of micrometastases to reduce systemic recurrence. Practically, this approach additionally ensures that treatment commences early whilst radiotherapy is planned. We hypothesised that it also provides a window of opportunity in which to assess treatment response in real time, such as via assessment of metabolic response, and switch to an alternative systemic agent where required.

There is existing prospective evidence that supports consideration of this approach in OAC.^15^^,^^16^^,^^19^^,^^29^, ^30^, ^31^ This includes the non-randomised, single-centre MUNICON I & II studies, which hypothesised that proceeding directly to surgery or to naCRT based on PET-CT assessed poor metabolic response to a first cycle of neoadjuvant chemotherapy might improve outcomes, albeit in the context of a number of now well-documented limitations.^32^ The more recent AGITG DOCTOR trial expanded on MUNICON I, demonstrating that a greater than 35% SUV_max_ decrease at day 15 of cycle 1 of neoadjuvant cisplatin/5-FU was associated with favourable overall and PFS in patients with OAC.^16^^,^^31^ The addition of docetaxel alone to metabolic non-responders in that trial improved pathological response rates but not survival, whereas addition of docetaxel with chemoradiation improved pathological response, PFS and locoregional recurrence, matching the metabolic-responder group. The recent MEMORI trial has in turn expanded on MUNICON II, demonstrating that PET-CT assessment after cycle 1 of neoadjuvant chemotherapy is prognostic for outcome in OAC, though here a switch to CROSS-regimen CRT in those not responding to neoadjuvant chemotherapy did not improve the primary outcome R0 resection rate measure.^15^^,^^29^

Similar to our approach, the CALGB80803 study proposed that a PET-CT directed change in systemic therapy based on response to induction treatment could improve outcomes in OAC patients receiving neo-adjuvant CRT followed by surgery.^19^ To achieve this, the investigators randomised patients to receive induction oxaliplatin, leucovorin and fluorouracil (FOLFOX) or carboplatin/paclitaxel. Non-responders, defined as a <35% decrease in SUV_max_ on PET-CT, were switched to the alternative chemotherapy option for naCRT. This study demonstrated that PET response was prognostic for survival outcomes (48.8 months vs 27.4 months). For patients who started on car/pac chemotherapy, non-responders who switched to FOLFOX during naCRT (pCR 20%) had comparable response rate to responders who continued car/pac (pCR 14%), however for patients who started on FOLFOX, responders who continued the same chemotherapy had much better pCR (40%) than non-responders who switched to car/pac (18%).

There is controversy regarding the extent to which pCR translates to survival outcomes in OAC.^33^ The study reported here used 24-week TFFS, which is known to correlate with survival, as on outcome measure.^5^ It is, therefore, disappointing that the premature close of this study arm means that we are unable to provide further evidence relating to the utility of PET-CT in directing the systemic arm of CRT in patients with OAC. We are nevertheless able to provide evidence that indicates that there is no improvement in outcomes with the use of early metabolic response assessment in patients with OSCC. This has not previously been subject to prospective assessment, though a previous retrospective analysis focussed solely on OSCC predicted the results we have outlined here.^34^ PET-CT nevertheless remains prognostic for outcome in CRT-treated OSCC patients, as was previously proposed for both OAC and OSCC in a retrospective analysis by Ilson and colleagues.^35^

It is unclear why PET-CT directed systemic switch appears to hold some promise in OAC but not in OSCC. It may, though, reflect the lower intrinsic radiosensitivity and higher propensity for systemic relapse of OAC; both of which necessitate optimal use of systemic agents. It may also relate to the choice of alternative chemotherapy for non-responders used in this study. It is, for instance, noteworthy that a switch to car/pac achieved significantly worse TFFS and overall survival in patients with OSCC, and inferior TFFS in those with OAC. At 78.6 and 92.6% for OSCC and 77.8 and 80.0% for OAC in responders and non-responders respectively, TFFS with cis/cap exceeded that seen in comparable previous studies of dCRT such as SCOPE1 (76.9%).^6^ In contrast, the TFFS of 68.0% and 66.7% for OSCC and OAC seen with car/pac is comparable, if not inferior, to current outcomes seen in the literature. Whilst this is somewhat supported by the improved outcomes seen with a similar FOLFOX regimen for patients receiving dCRT for OAC in the CALGB80803 trial, long-term data from the NeoSCOPE trial suggest superior overall survival with car/pac compared with oxaliplatin and capecitabine in patients receiving neoadjuvant CRT for OAC.^19^^,^^36^ However, it should be noted that in ART-DECO (OSCC 61%, 39% OAC), a dCRT trial which used car/pac as systemic, survival was lower than SCOPE1 which used cis/cap regimen therapy (3 year OS ∼40% vs ∼50%).^22^ These data do suggest that the use of cis/cap may be superior, at least when compared with a switch to car/pac, particularly in OSCC.

Given that this trial was stopped early and did not reach its target size, it is also possible that these differences arise through random variation. The achieved sample size in OAC was particularly small and no reliable conclusions can be drawn. Equally, these results may reflect an as yet unclear interaction between cis/cap and higher-dose EBRT, particularly considering that a larger proportion of patients received higher-dose CRT in the cis/cap group. However, subgroup analysis in each RT dose group showed the same direction of effect (data not shown) and the regression model that included RT dose showed the same significant effect.

Endoscopic biopsy at 24 weeks was a protocol assessment but where it was not done, CT scan result only was used to judge the primary endpoint. This was allowed to enable a feasible recruitment target following our experience in SCOPE1 when endoscopy was missed for a variety of pragmatic clinical reasons e.g. patient too ill/declined and during the COVID19 pandemic. However, as can be seen in Table 5, endoscopic biopsy was done in 21 + 23/22 + 27 = 90% of cases and showed the same trend of worse outcome in the patients who switched chemotherapy.

Only 16 of 29 participating centres enrolled patients into the PET-CT substudy due to concerns relating to funding for the additional day 14 response assessment imaging, which may have resulted in selection bias. There has also been no central review of the PET-CT data and randomisation was based on local assessment of SUV_max_. However, PET-CT response assessment was undertaken using an unchanged scanner for each patient, a trial manual standardised image acquisition and reporting, and a previous study has shown high concordance between local and central FDG-SUV quantification.^30^ The optimum timing of PET-CT and parameter (e.g. SUV_max_ or Total Glycolytic Index) for a metabolic response assessment has yet to be defined and will vary depending on tumour type and chemotherapy regime. Furthermore, PET technology is rapidly evolving. Although all the scans performed in this study were on state of the art, time of flight PET-CT scanners, PET is now rapidly transitioning to digital scanners with markedly improved sensitivity and resolution, which, combined with improved PET image reconstruction, motion correction and AI (Artificial Intelligence) image enhancement will significantly increase the clinical utility of PET, particularly regarding small abnormalities, and therefore potentially improve the predictive value of response assessment PET-CT.^37^

These data suggest that metabolic response based on a reduction in PET-CT assessed SUV_max_ of at least 35% from baseline to day 14 of cycle 1 of induction chemotherapy is not associated with improved outcomes in patients with OSCC receiving dCRT. A switch from cis/cap to car/pac in metabolic non-responders at this timepoint results in inferior outcomes and cannot be recommended. Sample size was too small to draw conclusions in the OAC cohort. Whereas the early detection of ineffective treatments is critical in personalising management, further studies are required to determine whether PET-CT has any role in this setting.

## Contributors

TC, CH, SM, MH, and GG were involved in the design and development of the trial. TC, CH, SM, MH, RA, LN, SB, SG, SGw, GR, TM, HS, RR, AB, SF, ST were involved in the writing and review of the protocol. LN, SB, PM, SS, CH, RA were involved in day to day running of the trial and all authors contributed to the Trial Management Group. CH and SS were responsible for the statistical analysis. TC, CH, MH, CMJ and SM were responsible for preparing the manuscript. All authors have contributed to and approved the final draft.

## Data sharing statement

The Centre for Trials Research is a signatory of AllTrials and aims to make its research data available wherever possible, subject to regulatory approvals, any terms and conditions placed upon us from external providers, patient confidentiality and all laws concerning the protection of personal information. De-identified participant data and study documents (such as the study protocol, statistical analysis plan, participant information sheet, and informed consent form) are generally freely available, but recipients are expected to acknowledge the original creators in any public use of the data or in publishing research results based wholly or in part upon the data – anyone requesting access to data will be asked to agree to the terms of the Creative Commons Attribution 4.0 license. We may ask the requestor to cover reasonable cost for preparing and providing the data (for example physical storage and postage, where dataset size makes it impractical to provide data by electronic means). Please send requests for access to our data to the open data team (CTRDataSampleRequests@cardiff.ac.uk) for assessment, providing:

• sufficient detail to uniquely identify the dataset sought.
• appropriate contact details for the requestor.

## Declaration of interests

SM declares research funding from Celgene. TM declares consulting fees from AstraZeneca and Teysuno; reports participation fees from Pierre Fabre and Pfizer; and has a role at Perspectum. All other authors declare that they have no relevant conflicts of interest.

## Appendix A Supplementary data

Supplementary material

SCOPE2 PET Imaging Manual

Protocol
