# Supplementary material for: Efficacy of early PET-CT directed switch to carboplatin and paclitaxel based definitive chemoradiotherapy in patients with oesophageal cancer who have a poor early response to induction cisplatin and capecitabine in the UK: a multi-centre randomised controlled phase II trial
Source: eClinicalMedicine. 2023 Jun 26;61:102059. doi: 10.1016/j.eclinm.2023.102059 (PMC10318451; doi:10.1016/j.eclinm.2023.102059)
Supplement: Supplementary material [file mmc1.pdf]

SCOPE2 PET Imaging Manual

SCOPE 2

PET Imaging Manual

1. CONTACT LIST

For questions regarding the details outlined within this imaging manual, please contact the Trial Manager as outlined below. All general study related questions should be directed to the primary investigator at the recruiting site.

Please contact the following personnel for enquiries relating to:	Contact
PET Expert for trial	**Kevin Bradley -**<br><br>Director of Clinical PET Research<br>PETIC<br>University Hospital of Wales<br>Cardiff.<br>CF14 4XW<br>Email: BradleyK2@cardiff.ac.uk
Quality Control and Data Transfer of PET-CT	**Core Lab Physicist**<br><br>UK PET Core Lab,<br>PET Centre, First Floor, Lambeth Wing, St Thomas' Hospital,<br>London, SE1 7EH<br>Telephone: 0207 188 7446 / 7445 / 1489<br>Email: pet-trials@kcl.ac.uk
Any other enquires	**Trial Manager**<br><br>Sarah Bridges<br>Telephone: 02920687201<br>Fax: 02920 687501<br>Email: SCOPE2@cardiff.ac.uk

© 2021 UK PET Core Lab

2. PET CENTRE ACCREDITATION PROCEDURE

The PET sub-study is now closed to recruitment in SCOPE2. PET-CT Site Accreditation and a Research ARSAC licence are no longer required for sites, but the following information about the accreditation process is included for information. For any sites wishing to go through the accreditation process to enable best practice, we will continue to offer support and guidance.

2.1 PET-CT Site Accreditation Process

The PET sub-study was suspended in August 2021 following the advice of the IDMC. As a consequence, PET centre site accreditation is no longer a requirement of the trial.

While the PET sub-study was open, a PET centre needed to undergo the PET site accreditation process before it could participate in the PET sub-study. Further details and written procedures for the site accreditation process were provided by the UK PET Core Lab, based at St Thomas' Hospital, London. Sites were required to contact the UK PET Core Lab (pet-trials@kcl.ac.uk) at an early stage to determine the requirements for the accreditation procedure.

For PET sub-study, no patients were to be scanned until all of the following steps had been completed:

1. Written confirmation from the UK PET Core Lab that scanning could start at your centre.
2. Confirmation of the ARSAC licence to be sent to the trial team at SCOPE2@cardiff.ac.uk or Centre For Trials Research, 6th Floor, Neuadd Meirionnydd, Heath Park, Cardiff, CF14 4YS.

Site Accreditation Overview

Accreditation of PET scanning centres was in place to set up and ensure the following:

- Named persons (and their deputies) with responsibility for scanning, QC and data transfer were identified at participating PET-CT centres.
- A tested and secure method was established at each site to transfer anonymised scan data between scanning facilities and the UK PET Core Lab and the central reporting facility.
- All image files were compliant with DICOM PART 10 format
- All image files were correctly anonymised and clearly named using pre-arranged file naming conventions.
- Image quality was comparable between centres, and standardised uptake values could be reliably determined from the PET-CT images.
- The proposed data acquisition/reconstruction protocol (including details of the time per bed position, CTAC parameters, reconstruction parameters etc) were agreed with the UK PET Core Lab before scanning started.

© 2021 UK PET Core Lab

PET scanning centres had to also repeat the site accreditation process in the following situations:

- After any software or hardware changes which may affect the scanner image quality.
- If there were any significant changes to the acquisition or reconstruction parameters originally agreed with the UK PET Core Lab
- Any other circumstances that arose that the Core Lab deemed may alter the image quality such as; QC failures, apparent scanner degradation or poor image quality.
- It was the responsibility of the participating PET centre to inform the Core Lab of any upgrades to the scanner hardware or software prior to the upgrade. If the upgrade was likely to affect the image quality the site would be required to repeat the phantom scans before continuing to scan patients as part of the trial.

2.2 Quality Control (QC) Procedures

We acknowledge that baseline PET-CT scans are not acquired as trial procedures so may have taken place on scanners without research accreditation. Wherever possible we would like to obtain confirmation of quality for the future use and publication of the trial data. Therefore we recommend the following:

PET-CT Scanners

A documented PET-CT scanner quality assurance program should be in place and records kept, covering daily, monthly, quarterly and annual QC testing.

The PET scanner should have up-to-date calibration and normalization. On the day of scanning a trial patient the manufacturer's recommended daily QC should be performed and if any failures or abnormalities are identified that could affect the quality of the PET scan; consideration should be given to rescheduling the scan.

Routine CT QC should be performed according to the manufacturer's recommendations, and should include a water filled phantom scanned on a weekly basis, to measure image noise and CT number as described in IPEM (Institute of Physics and Engineering in Medicine) report 91.

All PET-CT scanners to be used for the trial should be calibrated against the institutions own radionuclide calibrator which should have an 18F factor traceable to a primary standard.

Ancillary Equipment

As this study uses Standardised Uptake Values (SUV) defined in terms of patient weight, the scales used to weigh the patients must be calibrated. As a minimum the scales must be checked using a standard weight at least annually and should be accurate to within ±1kg of a standard weight of 70 kg and records kept.

The BM glucometer QC should be performed according to the manufacturer's or institution's procedure to ensure proper functioning.

© 2021 UK PET Core Lab

Quality assurance procedures for the radionuclide calibrator must be in place and activity measurements for ^{18}F should be traceable to a primary standard. QC tests should include daily constancy checks and annual accuracy and linearity.

Clocks used to record the assay time and injection time must be synchronized to the scanner time.

Do not take blood samples for evaluation after the injection of the isotope for the rest of that day.

Additional Scanner QC Required During the Trial

Standardised Uptake Values (SUV) are used as a primary tumour response endpoint, therefore accurate and consistent estimation of SUV for all patient scans and between all participating centres is required. This will be achieved via a rigorous and regular testing of SUV accuracy and consistency of all participating scanners.

Ideally, a uniform phantom should be scanned prior to the start of each scanning session in which a patient is to be scanned as part of the trial. This can either be a resin ^{68}Ge phantom (where available) or an ^{18}F water filled phantom. The activity concentration in the ^{18}F phantom should be approximately 5kBq/ml. The average SUV for a large ROI placed at the centre of the phantom must be 1.00 ± 10% and ideally within ± 5%. On visual inspection of the PET and CT the images should show no artifacts. The relevant sections of the PET Acquisition Form (Appendix 1) must be completed to confirm the results of this test. If the uniform phantom scan is not performed on the day of the patient scan, the result from the most recent 18F uniform cylinder scan should be recorded on the PET Acquisition form with the date performed.

Confirmation of Site Approval

Formerly, while the PET sub-study was running, once all the above steps had been completed, the UK PET Core Lab would issue a letter giving approval to scan patients enrolled in the Trial. The letter would be forwarded to both the PET centre and the Trial Coordinator at the Centre For Trials Research to confirm that the centre could now participate in the trial.

Scanning sites were to inform the Core Lab of any upgrades to the scanner hardware or software prior to the upgrade. If the upgrade was likely to affect the image quality the site would be required to repeat the phantom scans before continuing to scan patients as part of the trial. Sites were to also notify the Core Lab immediately of any deviations in QC and scan acquisition or reconstruction parameters from those agreed.

3. IMAGING PROCEDURE

We acknowledge that baseline PET-CT scans are not acquired as trial procedures so may have taken place on scanners without research accreditation, but we recommend the following:

© 2021 UK PET Core Lab

3.1 *Imaging Acquisition*

Purpose of Document

The purpose of this document is to describe the procedures necessary to perform the PET-CT scans on patients potentially enrolled in the trial.

Health and Safety Considerations

Local health and safety requirements should be followed at all times.

Overview

The study will be conducted at centres across the UK and imaging using both fixed and mobile PET/CT scanners.

As we are no longer recruiting to the PET sub-study, no further day 14 scans will take place.

3.2 *PET-CT Scan Timing*

Baseline PET-CT Scan prior to randomisation as part of standard practice. This should be within 5 weeks of the date of enrolment, and within 6 weeks of the start of treatment. If outside of these time frames, please contact the SCOPE2 team on SCOPE2@cardiff.ac.uk to discuss.

3.3 *The Response PET-CT Scan at day 14 is no longer taking place in SCOPE2, but the following information is provided for reference. Recommendations for Patient Preparation and Scan Procedure*

- Non-diabetic patients should fast for at least 6 hours prior to the scan. Plain (unflavoured) water should be taken during the period of fasting and the uptake period to ensure good hydration.
- Diabetic patients should be given a late morning appointment.
- Patients on insulin should eat a normal breakfast and administer insulin as usual.
- Diabetic patients on oral medication should eat a normal breakfast at least six hours before the examination and take their usual oral medication to control the blood sugar.
- The blood glucose level of all patients should be measured on arrival at the imaging centre and recorded on the PET Acquisition Form (Appendix 1). This should be performed using a calibrated glucometer or similar bedside device. Consideration should be given to rescheduling the scan if BM measures > 11mM/l (>200mg/dl). Insulin should not be administered to reduce glucose level.
- Oral diazepam (5-10mg po) may be given if desired to reduce brown fat uptake 30-60 minutes prior to tracer injection.
- Prior to injection, patients should be weighed without shoes and coats using a calibrated device and this should be recorded on the PET Acquisition Form (Appendix 1).
- During the tracer administration and uptake phase, the patient should remain seated or supine.
- Patients should be asked to void immediately prior to the PET-CT scan to reduce

© 2021 UK PET Core Lab

bladder activity.

3.4 Procedure for ^{18}F-FDG PET-CT Scan

No further day 14 scans to take place as the PET sub-study is closed to recruitment. The following information is provided for reference only.

While the PET sub-study was open, sequential PET exams for a patient were ideally to be performed in the same centre using the same accredited PET-CT system as the baseline scan. The patient preparation, tracer administration, image acquisition and reconstruction for these scans were to be matched for each subsequent patient scan acquired for the study. If for any reason, a baseline PET scan had been performed already as standard of care prior to study registration on a non-approved scanner and/or using a different protocol to the trial, response scans were to be performed using the imaging procedures described in this manual.

Where scanning conditions were not matched between response and baseline scans, subset analysis may have been required for quantitative assessments.

Radiopharmaceutical Administration

Radiopharmaceutical:	^{18}F-Fluorodeoxyglucose (FDG)
Activity:	400MBq (±10%) or according to local protocol and may vary dependent on scanner settings and patient size. While PET sub-study was open, injected activity should have been matched for baseline and response scans
Route of Administration:	Intravenous administration via butterfly cannula under quiet conditions. Syringe residue should be measured and the corrected injected activity documented on the PET Acquisition Form (Appendix 1).

Uptake Period

- The PET acquisition should ideally be started 60 to 90 minutes after the dose administration. If the baseline scan is performed outside these times prior to registration in the trial, please record the actual uptake time on the PET acquisition form (Appendix 1).
- While the PET sub-study was open, the response scans should have been performed at the same time after injection as the baseline scan ± 10 minutes, but not less than 55 minutes post injection.

Scan Procedure

- Intravenous CT contrast media should not be administered prior to the PET study.
- If local protocols include a diagnostic CT scan using contrast as part of the PET-CT examination this should be performed after the PET scan. In this case a separate low dose CT without contrast should also be acquired before the PET acquisition and this scan should be used for attenuation correction of the PET images.

© 2021 UK PET Core Lab

- Patients will generally be positioned in the PET-CT scanner with their arms raised above the head. If participants cannot tolerate this position for the duration of the study, a different participant positioning may be chosen. However, arms should be positioned in the same way at the baseline and the follow-up studies.
- A low-dose CT scan will be acquired for attenuation correction and anatomical localization of findings in the PET scan.
- The PET and CT scans should include the region between the base of skull and mid-thigh.
- All other imaging parameters i.e. with regard to time per bed position, CTAC acquisition parameters must be agreed with the UK PET Core Lab prior to the start of the study (see Section 2). These should then be used throughout the study. Any changes to these parameters must be agreed with the Core Lab before scanning any more patients.

3.5 Image Reconstruction

The reconstructed PET data should be corrected for decay, dead time, scatter, randoms and attenuation using standard algorithms provided by the scanner manufacturers.

Attenuation correction should be performed using the low dose CT.

Iterative reconstruction should be used e.g. OSEM or similar.

Both attenuation-corrected and non-attenuation-corrected PET images should be reconstructed.

All other reconstruction parameters i.e. with regard to number of iterations or filtering parameters must be agreed with the UK PET Core Lab prior to the start of the study. These should then be used throughout the study. Any changes to these parameters must be agreed with the Core lab before scanning any more patients.

4. DATA TRANSFER AND STORAGE

Please note, although the PET sub-study is now closed, we are still requesting the transfer of baseline scan data for SCOPE2 patients.

4.1 Anonymisation and File Naming Procedure

All patient identifying information must be removed from the images prior to transfer.

PET-CT studies should be clearly named using the following filename convention:

SCOPE2_< Patient trial number >_< Patient initials >_baseline

SCOPE2_< Patient trial number >_< Patient initials>_Day14

4.2 Data Transfer and Archive

PET-CT data should be transferred to the UK PET Core Lab at the same time as the completed PET-CT acquisition form (Appendix 1). All studies to be transferred to the Core Lab must be compliant with DICOM PART 10 format. FUSED images, screen captures and dose reports are not required.

© 2021 UK PET Core Lab

The following DICOM files are required:

- CT attenuation corrected half body PET images (base of skull to mid-thigh)
- Non-attenuation corrected half body PET images
- Half body CT scan

Secure data transfer procedures will be established with each scanning site. For instructions on approved transfer methods please contact the UK PET Core Lab (pet-trials@kcl.ac.uk).

- The main method for sites to upload pseudo-anonymised data to the Core Lab is using MIM software©. Designated users at sites will need to sign up for a MIMcloud clinical user account, after which access will be granted to the Core Lab server. As an alternative for sites unable to use the MIM software, access will be granted to the University server and pseudo-anonymised data can be uploaded using the secure file transfer protocol (sftp). Instructions for both these methods will be provided by the UK PET Core Lab (pet-trials@kcl.ac.uk).

Alternatively, data can be sent on CD/DVD by post to:

UK PET Core Lab

PET Centre

1st Floor, Lambeth Wing

St Thomas' Hospital

Westminster Bridge Road

London SE1 7EH

It is strongly recommended that scans be sent by registered post (recorded or special delivery) or courier. These can be tracked on the Royal Mail/ courier website.

- All reconstructed CT, CT-attenuated PET and non-attenuated PET data must be saved locally on an approved data storage device. Raw PET data should be archived according to local policy, and at least until the images have been accepted by the UK PET Core Lab in case additional reconstructions are required.
- Following review, all scan data will be archived at the Centre For Trials Research, Cardiff University.

Information to be recorded for each patient

For each patient study, the PET-CT acquisition information must be recorded on the PET-CT Acquisition Form (Appendix 1) and sent to the UK PET Core Lab with the PET-CT images.

Please email that a scan has been sent with the PET acquisition form (pet-trials@kcl.ac.uk) to the UK PET Core Lab.

5. PET-CT IMAGE ANALYSIS

Technical QA/QC of individual PET scans will be undertaken by the UK PET Core Lab which is part of the NCRI PET Research Network and provides national accreditation for PET research trial studies.

© 2021 UK PET Core Lab

The SUV_{max} value at the baseline (and, formerly, for day 14 PET scans) are assessed locally and recorded on the Case Report Form by the research nurse.

© 2021 UK PET Core Lab

SCOPE2 PET Imaging Manual

Appendix 1 **PET ACQUISITION DATA FORM FOR PET-CT SCANS**

(to be completed by PET scanning facility)

PET/CT scan acquired at: (PET Centre)	

Patient Details:

Patient's trial number:			
Date of PET/CT scan:			
Study timepoint (circle as applicable):	Baseline / Day 14		
Patient's weight (kg):		Patient's height (cm):	
Patient fasted for at least 6 hours:	Yes / No / Unknown (please circle)<br>if no, please indicate how long the patient fasted for:		
Patient blood glucose (mmol/l):			

Tracer Administration:

Activity in syringe (MBq):		Time measured (hh:mm):	
Residual activity in syringe (MBq):		Time measured (hh:mm):	
Time of administration (hh:mm):			
Any deviations from the imaging protocol?		Yes / No / Unknown (please circle)	
If yes, please specify			

Scanning Procedure:

PET scanner used (manufacturer and model):	
PET acquisition start time (hh:mm):	
Time per bed (mins) (or scan speed in mm/s):	

Quality Control:

Daily QC results for day of scan passed?	Yes / No / Unknown (please circle)
Mean SUV measured for uniform cylinder:<br>Date performed:	
Any deviations from the acquisition/ reconstruction parameters agreed with the Core Lab?	Yes / No / Unknown (please circle)
If yes, please specify	

Form completed by: ____________________ Date: _____________

Completed forms should be return via email: pet-trials@kcl.ac.uk

© 2021 UK PET Core Lab
