# Supplementary material for: Efficacy of early PET-CT directed switch to carboplatin and paclitaxel based definitive chemoradiotherapy in patients with oesophageal cancer who have a poor early response to induction cisplatin and capecitabine in the UK: a multi-centre randomised controlled phase II trial
Source: eClinicalMedicine. 2023 Jun 26;61:102059. doi: 10.1016/j.eclinm.2023.102059 (PMC10318451; doi:10.1016/j.eclinm.2023.102059)
Supplement: Protocol [file mmc3.docx]

**Supplementary materials for: A multi-centre randomised phase II trial of early PET-CT directed switch to carboplatin and paclitaxel based definitive chemoradiotherapy in patients with oesophageal cancer who have a poor early response to induction cisplatin and capecitabine**

**Chemotherapy dose modifications**

For haematological toxicity, dose modifications were based on full blood count readings taken within three days of the start of each cycle. For patients receiving a platinum and fluoropyrimidine, full dose treatment was delivered where the absolute neutrophil count (ANC) and platelet count were at least 1x10^9^/l and 75x10^9^/l. Chemotherapy was stopped until counts recovered then re-started at a 25% dose reduction if the ANC fell to 0.5-1.0x10^9^/l, if the platelet count fell to 50-74x10^9^/l or if there had been an episode of neutropenic sepsis during the previous cycle. If the ANC reached less than 0.5x10^9^/l or the platelet count fell to less than 50x10^9^/l chemotherapy was stopped until counts recovered and then re-started at a 50% dose reduction.

For patients receiving carboplatin and paclitaxel, full-dose treatment was delivered at an ANC of at least 1.0x10^9^/l and a platelet count of 100x10^9^/l for induction treatment, and at an ANC of at least 1.0x10^9^/l and a platelet count of at least 75x10^9^/l for weekly concurrent treatment. A fall in ANC and platelet count during induction treatment to less than 1.0x10^9^/l and 100x10^9^/l respectively resulted in a delay in treatment until counts recovered, followed by a 25% dose reduction. Where one of the ANC or platelet count fell to less than 1.0x10^9^/l and 100x10^9^/l respectively during induction treatment, chemotherapy was paused until counts recovered then resumed at full dose if recovery occurred within a week, or at a 25% dose reduction if it did not. Where ANC or the platelet count respectively fell to less than 1.0x10^9^/l or to 25-75x10^9^/l weekly concurrent treatment was omitted and then recommenced at a 25% dose reduction once counts had recovered. A fall in the platelet count to less than 25x10^9^/l with any platelet count led to the omission of both paclitaxel and carboplatin, with both dose reduced by 50% for subsequent cycles.

Renal dose adjustments were based on glomerular filtration rate (GFR) at baseline and during treatment. Patients with a GFR of less than 30ml/min were not eligible for study participation. Cisplatin and capecitabine were given at full dose where the GFR respectively exceeded 60ml/min and at least 50ml/min. At 50-60ml/min, cisplatin was given at a 75% dose reduction. Capecitabine was dose reduced to 75% at a GFR of 30-49ml/min. The cisplatin dose was reduced to 50% at a GFR of 40-49ml/min or replaced with carboplatin (AUC 5), which was used in place of cisplatin where GFR was 30-39ml/min. No dose modification for renal function was required for 5-FU, or for carboplatin and paclitaxel.

Hepatic dose adjustments were made for capecitabine but not for other therapies. Treatment was interrupted where AST or ALT levels exceeded five times the upper limit of normal (ULN) and resumed once these returned to 2.5 times the ULN or lower, with any repeat rise in ALT or AST resulting in a dose reduction to 75%. Grade 2 neurotoxicity resulting from paclitaxel led to treatment interruption until toxicity fell to grade 0-1, followed by a dose reduction to 75%. Patients were removed from the trial if neurotoxicity at grades 3-4 occurred. Unexplained chest pain of any grade in patients receiving a fluoropyrimidine resulted in the discontinuation of that treatment. The development of grade 2 or 3 palmoplantar erythrodysaesthesia caused capecitabine to be held until its resolution, with the dose restarted at a respective 15% or 25% dose reduction.

The first occurrence of other non-haematological toxicities led to treatment interruption at grades 2-3 and discontinuation at grade 4. Treatment recommenced once a grade 0-1 toxicity was reached from an initial grade of 2-3, though at a 75% dose reduction for grade 3 toxicities. A second occurrence of the same toxicity led to a dose reduction to 75% and 50% respectively for grade 2 and 3 toxicities. A third occurrence of the same toxicity at grade 2 resulted in a dose reduction to 50%. A third occurrence at grade 3 or a fourth occurrence at grade 2 led to the discontinuation of treatment.

# Radiotherapy quality assurance

##

## 1 Pre-accrual quality assurance

### 1.1 Completion of an outlining exercise

In an effort to streamline RTTQA and reduce workload for the PI we have revised the requirements on pre-accrual outlining exercises. PIs who did not complete the NeoSCOPE pre-accrual outlining exercises must complete the following exercises:

- Firstly, the SCOPE2 lower-third outlining exercise should be submitted, using the 3DCT reference dataset supplied with the 4DCT outlining exercise. Do not proceed to completing the 4DCT outlining exercise at this stage.
- Once the 3DCT volumes have been approved by the RTTQA team, centres wishing to use 4DCT for lower-third patients should then complete the 4DCT outlining exercise, delineating the 4DCT volumes using the full 4DCT dataset. The original 3DCT GTV, CTVA, CTVC and CTVB can be re-used hopefully saving the outliner’s time.
- Centres whose submissions of the lower-third outlining exercise have had issues may be asked to additionally complete the 3DCT mid-third outlining exercise.

Outlines will be compared against a consensus reference volume (gold standard) derived from the outlines of TMG members. Criteria for satisfactory completion will be at the discretion of the RTTQA members of the TMG based on the requirements of the protocol and this guidance document. Please use the GTVs provided. Attention will be paid to correct interpretation of imaging and ability to follow the protocol to create CTV and PTV. Written feedback will be provided to all centres.

### 1.2 Completion of a planning exercise

All centers must successfully complete the IMRT/VMAT credentialing program through the National RTTQA group or equivalent.

Centres must also satisfactorily complete a planning exercise for the high dose arm (i.e., including PTV_6000) using a pre-outlined 3DCT DICOM dataset provided by the RTTQA group. A Plan Assessment form (PAF, available on the SCOPE2 page of the RTTQA website) should be completed and submitted at the same time.

Plans will be checked for consistency with this document’s instructions. Criteria for satisfactory completion will be at the discretion of the RTTQA members of the TMG. Written feedback will be provided to all centres.

### 1.3Production of a Radiotherapy Process Document

All centres who wish to participate in SCOPE2 should submit a Radiotherapy Process Document describing how trial patients will be scanned, planned and treated. These will be reviewed by RTQA members of the TMG and clarifications may be required. A template for this document will be provided.

If the centre’s radiotherapy process changes during on-trial recruitment they should submit an amended copy of the Radiotherapy Process Document to the RTTQA contact highlighting the changes. These changes must be approved before further patients are treated.

### 1.4 Completion of the SCOPE2 Facilities Questionnaire

All sites must complete the SCOPE2 Facilities Questionnaire.

**2 On-trial quality assurance**

The following radiotherapy planning data must be submitted for **each** SCOPE2 patient before they start radiotherapy:

- Planning CT, Structures, Plan and Dose in DICOM format.
- For patients outlined using 4DCT the full CT set (i.e., all respiratory phases) should be submitted.
- Diagnostic reports (EUS, CT, PET-CT).
- Plan Assessment Form (PAF)

The PI should approve the outlining and planning for each SCOPE2 patient. The TMG encourage internal peer-review of trial patient treatment outlines and plans within individual participating centres and for centres to contact the SCOPE2 RTTQA contact if there are concerns or queries regarding the trial protocol.

All data must be fully anonymised to include only the patient’s trial number, the trial name (SCOPE2), date of birth and initials and submitted securely following instructions on the SCOPE2 page of the RTTQA website. Non-anonymised or incomplete data will cause delays in the review.

### 2.1 Prospective Individual Case Review (Real-time review)

There will be prospective individual case review of the outline and plan for:

- the first patient case recruited at each centre
- the first case outlined using 4DCT at each centre
- the first high-dose case recruited at each centre
- **all** high-dose cases submitted up until the first SCOPE2 toxicity analysis assessment
- any additional patients if the RTTQA team have concerns

This outlining and planning assessment will be conducted in real-time (i.e., completed before the patient starts radiotherapy) and undertaken by the RTTQA members of the TMG. As such, prospective individual case review will require timely uploading of the data from the centres. The RTTQA contact will process the review within 3 working days of receipt. Early submission of the outlining data is strongly encouraged to allow adequate time for review prior to the start of radiotherapy planning. It is left to the centre’s discretion as to whether they wish to start the planning process with the pre-approval outlines while awaiting this feedback.

### 2.2 Timely retrospective review

A 10% sample of SCOPE2 patients will be selected for timely-retrospective review via centrally-administered block randomisation process. Outlines and plans should be submitted for review by the RTTQA team as soon as possible and the RTTQA team will give feedback to the centre within 2 weeks of the start of treatment. Only deviations from protocol considered a significant risk to the patient/trial outcome will require a change in treatment plan at this stage.

## 2.3 Replans

If a SCOPE2 patient’s radiotherapy requires replanning or adjusting (e.g., change in Monitor Units for one or more beam) please:

- Record the reasons for the replan on the patient’s Case Report Form (CRF)
- Submit the replan to RTTQA accompanied by an updated PAF

**Figure S1. Trial Schema**

**Patients with oesophageal cancer**

**Group A: Phase II in ADENOCARCINOMA (and undifferentiated)**

**Group B: Phase II/III in SQUAMOUS CELL CARCINOMA**

**Arm 1**

**1 cycle**

**carboplatin and paclitaxel (q 3 weeks)**

**then**

**RT 50Gy/25#**

**concomitant with weekly carbo/paclitaxel**

**(A, n=10: B, n=31)**

**Arm 3**

**1 cycle**

**carboplatin and**

**paclitaxel (q 3 weeks)**

**then**

**RT 60Gy/25#**

**Concomitant with weekly carbo/ paclitaxel**

**(A, n=10: B, n=31)**

**Follow up**

**End of treatment assessment: week 12**

**Post treatment toxicity review for all patients: week 15**

**Additional post treatment toxicity review for patients within the high RT dose arm: week 18 (second additional visit at week 21 if deemed necessary by the treating clinician)**

**Endoscopic assessment, biopsy and CT at 6 months**

**Subsequent follow up visits: 24 weeks and 9, 12, 16, 20 and 24 months after enrolment, and annually thereafter for up to 5 years post enrolment**

**Arm 2**

**3 cycles**

**cisplatin and**

**capecitabine/5-FU (q 3 weeks)**

**+**

**RT 50Gy/25#**

**concomitant with last 2 cycles**

**(A, n=62: B, n=189)**

**Arm 4**

**3 cycles**

**cisplatin and**

**capecitabine/5-FU (q 3 weeks)**

**+**

**RT 60Gy/25#**

**concomitant with last 2 cycles**

**(A, n=62: B, n=189)**

**PET Non-Responders**

**Randomise**

**ENROLMENT**

**Cycle 1 = 1 cycle cisplatin + capecitabine with PET scan at day 14**

**PET Responders/not eligilble for PET randomisation**

**Randomise**

**Figure S2. Distribution of SUV_max_ levels in failures and failure-free patients at 24 weeks by trial arm**

|  | **carboplatin+paclitaxel** | **cisplatin+capecitabine** |
| --- | --- | --- |
| Baseline SUV_max_ | 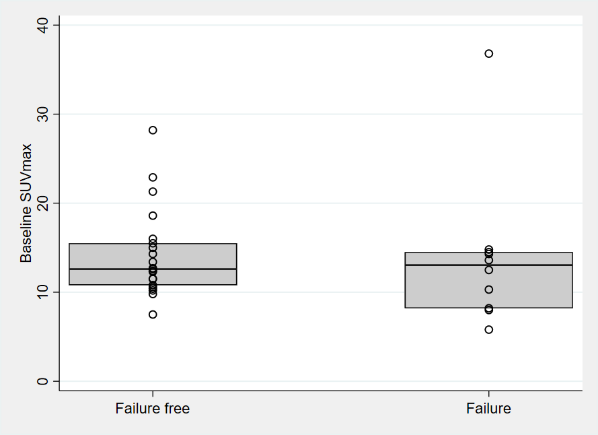  z = 0.613  Prob > \|z\| = 0.5400  Exact prob = 0.5533 | 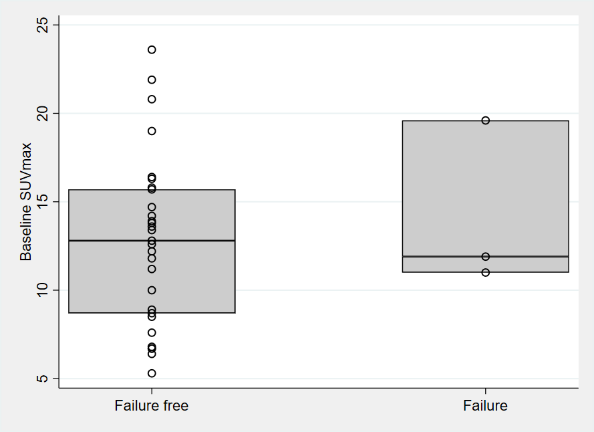  z = -0.291  Prob > \|z\| = 0.7711  Exact prob = 0.8048 |
| % change SUV_max_ (baseline to day 14) | 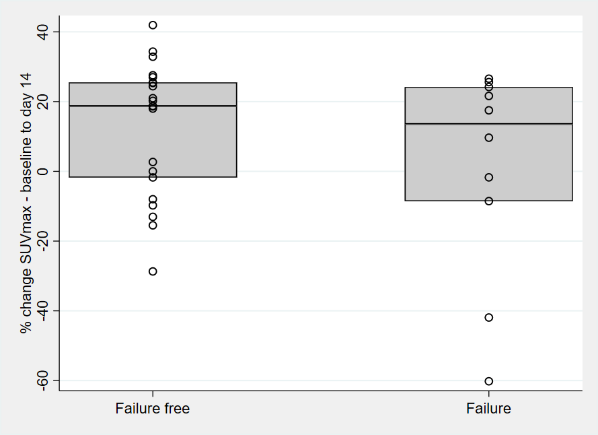  z = -0.887  Prob > \|z\| = 0.3749  Exact prob = 0.3927 | 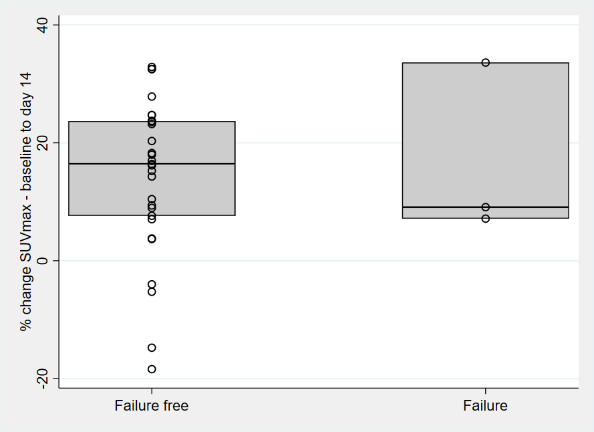  z = 0.097  Prob > \|z\| = 0.9227  Exact prob = 0.9516 |

**Figure S3. Overall survival by PET response**

**
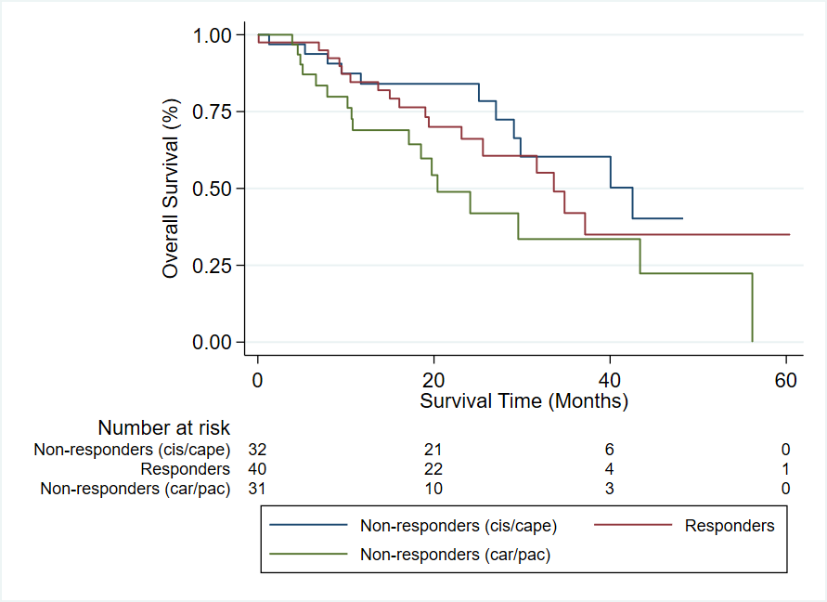
**

**Table S1. Inclusion and exclusion criteria for the SCOPE2 trial**

| **Inclusion criteria:**  Patients meeting the following criteria may be included in the trial:   1. 17 years of age or older. 2. Has been selected and is fit to receive potentially curative definitive chemoradiotherapy by a specialist Upper GI MDT*. 3. Histologically confirmed adenocarcinoma, undifferentiated cancer or squamous cell carcinoma. 4. Tumours of the cervical, thoracic oesophagus, or gastro-oesophageal junction (GOJ) with proximal extent of disease no more proximal than 15cm ab oral and distal extent of primary tumour no more than 2 cm beyond the GOJ. 5. Tumours staged as T1-4 and N+/-, as described in section 6.1. M1 nodes which are encompassible within the radical radiotherapy volume are eligible. 6. Total disease length (including primary tumour and involved lymph nodes) ≤13cm. The primary tumour should also be ≤10cm. 7. WHO performance status 0-1. 8. Adequate cardiovascular function for safe delivery of chemo-radiation in the opinion of the principal investigator. Where there is clinical concern patients should have an adequate cardiac ejection fraction ≥ 40% as determined by MUGA scan or ECHO (within 4 weeks prior to enrolment). 9. Adequate respiratory function for safe delivery of chemo-radiation in the opinion of the Principal Investigator. Where there is clinical concern FEV1 ≥ 1 litre as determined by spirometry (within 4 weeks prior to enrolment). 10. Patients may receive cisplatin and capecitabine (or 5FU) OR carboplatin and paclitaxel. For those patients selected for a platinum/fluoropyrimidine but deemed unfit for cisplatin (for example but not exclusively, due to advanced age, poor renal function, neurotoxicity or clinically significant hearing impairment), they may instead receive carboplatin (AUC5) upon the discretion of the treating clinician. 11. Patients with known DPD deficiency (partial or complete) may still be eligible for SCOPE2, provided they receive carboplatin and paclitaxel regimen throughout. 12. Adequate haematological, hepatic and renal function, measured within 2 weeks prior to enrolment.  - Absolute neutrophil count (ANC) ≥ 1.5x109/L - Platelets ≥ 100x109/L - Serum bilirubin ≤ 1.5x ULN - ALT / AST ≤ 2.5x ULN - ALP ≤ 3x ULN - Glomerular filtration rate ≥40 mls/min using locally agreed methodology.   - If GFR ≥60 mls/min by Cockroft-Gault or equivalent, dose modifications are not required.   - If GFR is 40 to 60 ml/min and cisplatin is to be given, then formal GFR estimation (EDTA, DTPA clearance or 24-hour clearance or local institutional equivalent) should be performed and the appropriate dose modifications for cisplatin used. Please note, if GFR is 40 to 60ml/min and carboplatin is to be given, formal GFR estimation is not mandatory.  1. Patients with reproductive potential (male or female), who are sexually active during the duration of the trial consent to using a highly effective method of contraception for at least six months after the last dose of chemoradiotherapy. 2. Patients who have provided written informed consent prior to enrolment.   Additional inclusion criteria for patient eligibility for the PET based randomisation (cisplatin/capecitabine vs carboplatin/paclitaxel) as assessed at local centre:   1. Baseline SUVmax ≥ 5 2. PET scan 14 days after start of chemo (-2/+3 days from this date is acceptable) 3. Less than 35% reduction in SUVmax on Day 14 PET (-2/+3 days) 4. Baseline PET-CT within 5 weeks prior to start date of treatment. 5. Patient consents to having additional PET scan. 6. Patient received the cisplatin/capecitabine regimen in cycle 1.   Patients who are ineligible for PET randomisation or who are unable//unwilling to have the additional PET will still be eligible for 50Gy vs 60Gy randomisation and will continue the same chemotherapy that they had in cycle 1. |
| --- |
| **Exclusion criteria:**  If any of the following criteria apply, patients cannot be included in the trial:   1. Patients who have had previous treatment for invasive oesophageal carcinoma or gastro-oesophageal junction carcinoma (not including PDT or laser therapy for high grade dysplasia/carcinoma in-situ). 2. Patients with metastatic disease. (unless M1 encompassable in the radical RT field) 3. Patients with other active malignancy or past malignancy which is deemed to have significant impact on their prognosis over the next three years. 4. Patients with >2cm mucosal extension of tumour into the stomach or where the superior extent is proximal to 15 cm ab oral. 5. Patients with unstable angina or uncontrolled hypertension or cardiac failure or other clinically significant cardiac disease. 6. Patients who need continued treatment with a contraindicated concomitant medication or therapy. 7. Patients with serious infections which in the opinion of the investigator make delivery of chemotherapy inappropriate. 8. Known hypersensitivity to IMPs. 9. Women who are pregnant or breastfeeding. 10. Patients with an oesophageal stent (patients requiring a PEG/RIG/feeding jejunostomy for nutritional purposes ARE eligible).   Any other situation, which in the opinion of the local PI, makes the patient an unsuitable candidate for this trial. |

**Table S2. Recruitment at the UK sites**

| **Study Site** | **n** | **%** |
| --- | --- | --- |
| Velindre Hospital | 9 | 8.74 |
| Southampton | 2 | 1.94 |
| Christie | 6 | 5.83 |
| Maidstone | 3 | 2.91 |
| Glan Clwyd | 8 | 7.77 |
| Singleton | 4 | 3.88 |
| Beatson | 5 | 4.85 |
| Coventry | 3 | 2.91 |
| Royal Marsden (London) | 3 | 2.91 |
| Royal Marsden (Sutton) | 8 | 7.77 |
| Churchill | 7 | 6.8 |
| Weston Park | 3 | 2.91 |
| Bristol | 9 | 8.74 |
| Raigmore | 1 | 0.97 |
| Castle Hill | 29 | 28.16 |
| Gloucestershire | 3 | 2.91 |
| **Total** | **103** | **100** |

**Table S3. 24 Week Treatment Failure-Free Survival (TFFS) at time of IDMC decision to stop randomisation**

|  | **Squamous cell carcinoma**  **(PET non-responders)** | | | | **Adenocarcinoma**  **(PET non-responders)** | | | |
| --- | --- | --- | --- | --- | --- | --- | --- | --- |
|  | **carboplatin+**  **paclitaxel** | | **cisplatin+**  **capecitabine** | | **carboplatin+**  **paclitaxel** | | **cisplatin+**  **capecitabine** | |
| Patients randomised | 25 | | 27 | | 6 | | 5 | |
|  | n | % | n | % | n | % | n | % |
| 24 wk not received yet | 5 | 20 | 3 | 11 | 2 | 33 | 0 | 0 |
| 24 week assessment valid* | 20 | 80 | 24 | 89 | 4 | 67 | 5 | 83 |
| Success | 14 | 70 | 22 | 92 | 3 | 75 | 4 | 80 |
| Failure | 6 | 30 | 2 | 8 | 1 | 25 | 1 | 20 |
| Endoscopy done | 16 | *80* | 19 | *79* | 3 | *75* | 3 | *60* |
| Success where endoscopy done | 13 | *81* | 18 | *95* | 3 | *100* | 3 | *100* |

*Died/progressed before 24 week CT scan due or CT scan done at 24 weeks (=/- 4 weeks)

**Table S4. Worst CTCAE grade of all toxicities between start of cycle 2 and week 24 (n (%))**

|  | **carboplatin+paclitaxel** | | | | **cisplatin+capecitabine** | | | |
| --- | --- | --- | --- | --- | --- | --- | --- | --- |
|  | **grade 1*** | **grade 2** | **grade 3** | **grade 4** | **grade 1*** | **grade 2** | **grade 3** | **grade 4** |
| **Blood and lymphatic system disorders** | | | | | | | | |
| Anaemia | 8 (26%) | 5 (16%) | 2 (6%) |  | 7 (23%) | 2 (6%) | 1 (3%) |  |
| Febrile neutropenia |  | 1 (3%) | 3 (10%) |  |  |  | 1 (3%) |  |
| **Cardiac disorders** | | | | | | | | |
| Arrythmia |  |  |  |  |  | 1 (3%) |  |  |
| Cardiac arrest |  |  |  | 1 (3%) |  |  |  |  |
| Conduction disorder / atrial fibrillation |  |  | 1 (3%) |  |  | 1 (3%) | 1 (3%) |  |
| Heart failure |  |  |  |  |  |  | 1 (3%) |  |
| **Ear and labyrinth disorders** | | | | | | | | |
| Hearing impaired | 6 (19%) | 1 (3%) | 1 (3%) |  | 3 (10%) | 1 (3%) |  |  |
| **Gastrointestinal disorders** | | | | | | | | |
| Abdominal pain | 3 (10%) | 3 (10%) |  |  | 3 (10%) |  | 1 (3%) |  |
| Colonic perforation |  |  |  |  |  |  | 1 (3%) |  |
| Constipation | 8 (26%) | 4 (13%) |  |  | 9 (29%) | 9 (29%) |  |  |
| Diarrhoea | 9 (29%) | 2 (6%) | 1 (3%) |  | 4 (13%) | 5 (16%) | 1 (3%) |  |
| Dry mouth |  |  |  |  | 2 (6%) |  |  |  |
| Dysphagia | 5 (16%) | 7 (23%) | 12 (39%) | 2 (6%) | 6 (19%) | 11 (35%) | 11 (35%) |  |
| Gastritis / ulcer |  | 1 (3%) |  |  |  |  |  |  |
| Gastroesophageal re-flux | 8 (26%) | 3 (10%) |  |  | 2 (6%) | 4 (13%) |  |  |
| Mucositis oral | 4 (13%) | 2 (6%) |  |  | 6 (19%) |  |  |  |
| Nausea / vomiting | 8 (26%) | 5 (16%) | 1 (3%) |  | 5 (16%) | 10 (32%) | 3 (10%) |  |
| Oesophageal pain | 9 (29%) | 10 (32%) |  |  | 7 (23%) | 6 (19%) | 1 (3%) |  |
| Oesophageal stenosis |  | 1 (3%) |  |  |  | 1 (3%) | 1 (3%) |  |
| Oesophagitis | 5 (16%) | 9 (29%) | 3 (10%) |  | 3 (10%) | 11 (35%) | 7 (23%) |  |
| Oral thrush |  | 1 (3%) |  |  |  | 2 (6%) |  |  |
| Stomatitis | 3 (10%) |  |  |  |  |  |  |  |
| Stricture |  |  |  |  | 2 (6%) |  |  |  |
| **General disorders and administration site conditions** | | | | | | | | |
| Fatigue | 6 (19%) | 14 (45%) | 2 (6%) |  | 8 (26%) | 18 (58%) | 1 (3%) |  |
| Fever | 2 (6%) | 1 (3%) |  |  | 3 (10%) |  | 1 (3%) |  |
| Peripheral oedema |  | 1 (3%) |  |  |  | 2 (6%) |  |  |
| **Immune system disorders** | | | | | | | | |
| ALT increased | 2 (6%) |  |  |  | 5 (16%) |  |  |  |
| AST increased |  |  |  |  | 2 (6%) |  |  |  |
| Allergic reaction | 2 (6%) | 2 (6%) |  |  |  |  |  |  |
| Bilirubin increased | 3 (10%) | 1 (3%) |  |  | 3 (10%) | 1 (3%) |  |  |
| Creatinine increased | 3 (10%) |  |  |  |  | 1 (3%) |  |  |
| Neutrophil decreased | 3 (10%) | 5 (16%) | 4 (13%) | 1 (3%) | 6 (19%) | 1 (3%) | 4 (13%) | 1 (3%) |
| Platelet decreased | 7 (23%) | 4 (13%) | 1 (3%) |  | 13 (42%) | 2 (6%) | 2 (6%) |  |
| Weight loss | 5 (16%) | 4 (13%) |  |  | 8 (26%) | 4 (13%) | 1 (3%) |  |
| **Infections and infestations** | | | | | | | | |
| Chest infection |  |  | 1 (3%) |  |  | 2 (6%) | 1 (3%) |  |
| Infection (other) |  |  | 1 (3%) |  |  |  | 2 (6%) |  |
| Sepsis |  |  |  |  |  |  | 1 (3%) |  |
| Skin infection |  |  | 2 (6%) |  |  |  |  |  |
| Urinary tract infection |  | 2 (6%) |  |  |  |  |  |  |
| **Injury, poisoning and procedural complications** | | | | | | | | |
| Dermatitis radiation | 2 (6%) |  |  |  |  |  |  |  |
| Radiation lung injury |  | 1 (3%) |  |  |  |  |  |  |
| **Investigations** | | | | | | | | |
| Hypoalbuminaemia |  | 1 (3%) |  |  |  | 1 (3%) |  |  |
| Lymphocyte count decreased |  |  |  | 1 (3%) |  | 1 (3%) | 1 (3%) |  |
| WBC decreased |  | 2 (6%) |  |  | 3 (10%) | 1 (3%) |  |  |
| **Metabolism and nutrition disorders** | | | | | | | | |
| Anorexia | 5 (16%) | 7 (23%) | 1 (3%) |  | 2 (6%) | 6 (19%) | 3 (10%) |  |
| Dehydration |  |  | 2 (6%) |  |  |  | 2 (6%) |  |
| Hyperuricemia | 2 (6%) |  |  |  | 6 (19%) | 1 (3%) |  |  |
| Hypokalaemia | 7 (23%) | 1 (3%) | 1 (3%) |  | 4 (13%) |  | 2 (6%) |  |
| Hypomagnesemia | 7 (23%) | 2 (6%) |  |  | 4 (13%) | 1 (3%) | 1 (3%) |  |
| Hypophosphataemia |  |  |  |  |  | 1 (3%) |  |  |
| **Musculoskeletal and connective tissue disorders** | | | | | | | | |
| Arthralgia | 4 (13%) | 1 (3%) |  |  |  |  |  |  |
| Back pain | 2 (6%) |  |  |  |  | 1 (3%) |  |  |
| Peripheral pain (other) |  | 1 (3%) |  |  |  |  |  |  |
| Weakness |  | 1 (3%) |  |  |  | 1 (3%) |  |  |
| **Neoplasms benign, malignant and unspecified (incl. cysts and polyps)** | | | | | | | | |
| Ganglion cyst |  | 1 (3%) |  |  |  |  |  |  |
| **Nervous system disorders** | | | | | | | | |
| Dizziness | 2 (6%) |  |  |  | 2 (6%) | 1 (3%) |  |  |
| Dysgeusia |  | 2 (6%) |  |  | 3 (10%) | 1 (3%) |  |  |
| Headache | 2 (6%) |  |  |  |  |  |  |  |
| Peripheral motor neuropathy | 2 (6%) |  |  |  |  |  |  |  |
| Peripheral sensory neuropathy | 5 (16%) | 1 (3%) |  |  | 4 (13%) | 1 (3%) |  |  |
| Stroke |  |  |  |  |  |  | 1 (3%) | 1 (3%) |
| Syncope |  |  | 1 (3%) |  |  |  |  |  |
| **Psychiatric disorders** | | | | | | | | |
| Acute confusion |  |  | 1 (3%) |  |  |  |  |  |
| Anxiety |  | 1 (3%) |  |  |  | 1 (3%) |  |  |
| Insomnia |  | 1 (3%) |  |  |  |  |  |  |
| **Renal and urinary disorders** | | | | | | | | |
| Urea increased |  |  |  |  | 2 (6%) |  |  |  |
| **Respiratory, thoracic and mediastinal disorders** | | | | | | | | |
| Aspiration |  |  |  |  |  |  | 2 (6%) |  |
| Chest pain | 4 (13%) |  |  |  |  | 1 (3%) |  |  |
| Cough | 11 (35%) |  |  |  | 8 (26%) | 1 (3%) |  |  |
| Dyspnoea | 7 (23%) | 1 (3%) | 1 (3%) |  | 7 (23%) | 2 (6%) | 1 (3%) |  |
| Epistaxis | 2 (6%) |  |  |  |  |  |  |  |
| Pneumonitis |  |  | 1 (3%) |  |  |  |  |  |
| Pulmonary oedema |  |  |  |  |  | 1 (3%) |  |  |
| Respiratory (other) |  |  |  |  |  | 1 (3%) |  |  |
| Sore throat |  |  |  |  | 2 (6%) |  |  |  |
| **Skin and subcutaneous tissue disorders** | | | | | | | | |
| Alopecia | 3 (10%) | 11 (35%) | 1 (3%) |  | 6 (19%) |  |  |  |
| Dry skin | 3 (10%) | 1 (3%) |  |  | 4 (13%) |  |  |  |
| PPE syndrome | 4 (13%) |  |  |  | 5 (16%) | 1 (3%) |  |  |
| Rash (other) |  |  |  |  |  | 1 (3%) |  |  |
| Rash maculo-papular | 3 (10%) |  |  |  | 2 (6%) |  |  |  |
| **Vascular disorders** | | | | | | | | |
| Hypotension |  | 1 (3%) | 1 (3%) |  | 4 (13%) | 2 (6%) | 1 (3%) |  |
| Thromboembolic events |  | 2 (6%) |  |  |  | 1 (3%) | 1 (3%) | 1 (3%) |

*Only grade 1 toxicities occurring in >5% of patients are included

**Table S5. Univariable and multivariable cox regression analysis of OS**

|  |  | **OS (months)** | | | **Univariable** | | | **Multivariable** | | |
| --- | --- | --- | --- | --- | --- | --- | --- | --- | --- | --- |
|  |  | n | Median | 95% CIs | HR | 95% CIs | p | HR | 95% CIs | p |
| Trial arm | Car/Pac | 31 | 20.4 | 10.8-43.4 |  |  |  |  |  |  |
|  | Cis/Cap | 32 | 42.5 | 27.0-nr | 0.44 | 0.20-0.97 | 0.041 | 0.36 | 0.16-0.84 | 0.018 |
| Age | <70 | 32 | 29.6 | 11.7-nr |  |  |  |  |  |  |
|  | ≥70 | 31 | 40.0 | 24.1-nr | 0.61 | 0.28-1.32 | 0.207 | 0.50 | 0.22-1.13 | 0.095 |
| Sex | Male | 33 | 40.0 | 20.4-nr |  |  |  |  |  |  |
|  | Female | 30 | 29.6 | 18.5-nr | 0.97 | 0.45-2.11 | 0.939 | 0.70 | 0.29-1.74 | 0.433 |
| WHO status | 0 | 31 | 43.4 | 29.6-nr |  |  |  |  |  |  |
|  | 1 | 32 | 27.0 | 17.1-nr | 2.41 | 1.06-5.48 | 0.036 | 2.96 | 1.19-7.38 | 0.020 |
| Stage | I or II | 25 | 42.5 | 25.1-nr |  |  |  |  |  |  |
|  | III | 38 | 29.6 | 19.7-nr | 1.35 | 0.62-2.95 | 0.454 | 1.23 | 0.50-3.03 | 0.652 |
| Tumour type | Squamous | 52 | 29.8 | 24.1-nr |  |  |  |  |  |  |
|  | Adeno/undiff | 11 | 42.5 | 3.91-nr | 1.12 | 0.45-2.80 | 0.809 | 0.86 | 0.31-2.41 | 0.773 |
| Total disease length | <5cm | 24 | 29.6 | 19.7-nr |  |  |  |  |  |  |
|  | ≥5cm | 39 | 40.0 | 20.4-nr | 1.15 | 0.51-2.59 | 0.729 | 1.20 | 0.46-3.09 | 0.708 |

nr=not reached

**Table S6. Cause of death**

|  | **carboplatin+paclitaxel** | **cisplatin+capecitabine** |
| --- | --- | --- |
| Oesophageal cancer | 10 | 8 |
| Myocardial infarction | 0 | 2 |
| Cardiac arrest | 2 | 0 |
| Bronchial pneumonia | 0 | 1 |
| Progression of new primary | 1 | 0 |
| Pneumonitis | 1 | 0 |
| Sepsis due to cholecystitis | 1 | 0 |
| Complications from salvage surgery | 1 | 0 |
| Fall | 1 | 0 |
| **Total** | **17** | **11** |

**Table S7. Univariable and multivariable cox regression analysis of PFS**

|  |  | **PFS (months)** | | | **Univariable** | | | **Multivariable** | | |
| --- | --- | --- | --- | --- | --- | --- | --- | --- | --- | --- |
|  |  | n | Median | 95% CIs | HR | 95% CIs | p | HR | 95% CIs | p |
| Trial arm | Car/Pac | 31 | 19.4 | 10.8-24.1 |  |  |  |  |  |  |
|  | Cis/Cap | 32 | 34.6 | 18.3-nr | 0.54 | 0.27-1.08 | 0.079 | 0.58 | 0.28-1.21 | 0.147 |
| Age | <70 | 32 | 20.1 | 10.8-nr |  |  |  |  |  |  |
|  | ≥70 | 31 | 24.1 | 18.5-nr | 0.74 | 0.37-1.49 | 0.396 | 0.58 | 0.27-1.22 | 0.152 |
| Sex | Male | 33 | 20.6 | 12.3-43.4 |  |  |  |  |  |  |
|  | Female | 30 | 24.1 | 18.3-nr | 0.81 | 0.40-1.64 | 0.565 | 0.72 | 0.33-1.55 | 0.398 |
| WHO status | 0 | 31 | 34.6 | 20.1-nr |  |  |  |  |  |  |
|  | 1 | 32 | 19.4 | 18.7-nr | 1.65 | 0.81-3.35 | 0.164 | 1.90 | 0.87-4.16 | 0.109 |
| Stage | I or II | 25 | 21.4 | 17.1-nr |  |  |  |  |  |  |
|  | III | 38 | 20.1 | 10.8-nr | 1.17 | 0.64-2.64 | 0.474 | 1.02 | 0.58-2.36 | 0.656 |
| Tumour type | Squamous | 52 | 24.1 | 18.5-nr |  |  |  |  |  |  |
|  | Adeno/undiff | 11 | 13.1 | 3.9-nr | 1.75 | 0.79-3.90 | 0.171 | 1.62 | 0.66-3.95 | 0.293 |
| Total disease length | <5cm | 24 | 20.1 | 17.1-nr |  |  |  |  |  |  |
|  | ≥5cm | 39 | 24.1 | 10.8-nr | 0.99 | 0.48-2.03 | 0.969 | 1.00 | 0.44-2.31 | 0.997 |

nr=not reached

**Table S8. Patterns of first progression in relation to radiation target volumes by tumour type and trial arm (number of events)**

|  | **carboplatin+paclitaxel** | | | | **cisplatin+capecitabine** | | | |
| --- | --- | --- | --- | --- | --- | --- | --- | --- |
|  | **Infield** | **Outfield** | **Both** | **Unknown** | **Infield** | **Outfield** | **Both** | **Unknown** |
| **Squamous cell carcinoma** | | | | | | | | |
| Death |  |  |  | 8 |  |  |  | 3 |
| Loco-regional only | 2 | 1 | 0 | 0 | 2 | 0 | 0 | 2 |
| Loco-regional plus distant | 0 | 0 | 0 | 2 | 0 | 0 | 0 | 0 |
| Distant only |  | 4 |  |  |  | 2 |  |  |
| **Total** | **2** | **5** | **0** | **10** | **2** | **2** | **0** | **5** |
| **Adenocarcinoma/undifferentiated** | | | | | | | | |
| Death |  |  |  | 3 |  |  |  | 0 |
| Loco-regional only | 0 | 0 | 0 | 0 | 2 | 1 | 0 | 0 |
| Loco-regional plus distant | 0 | 0 | 0 | 0 | 0 | 0 | 0 | 1* |
| Distant only |  | 0 |  |  |  | 1 |  |  |
| **Total** | **0** | **0** | **0** | **3** | **2** | **2** | **0** | **1** |

*this patient progressed prior to start of radiotherapy

**Table S9. Baseline patient characteristics by PET response**

|  | **PET non-responders cisplatin+capecitabine** | | **PET responders** | |  |
| --- | --- | --- | --- | --- | --- |
| **Number enrolled** | 32 | | 40 | |  |
| **Median age (years)**, IQR | 70.3 (61.1-72.1) | | 70.6 (63.8-76.8) | |  |
| **Gender** |  |  |  |  |  |
| Male | 19 | (59.4) | 22 | (55.0) |  |
| Female | 13 | (40.6) | 18 | (45.0) |  |
| **WHO Performance Status** |  |  |  |  |  |
| 0 | 19 | (59.4) | 26 | (65.0) |  |
| 1 | 13 | (40.6) | 14 | (35.0) |  |
| **Reason for non-surgical therapy** |  |  |  |  |  |
| Clinician's choice | 17 | (53.1) | 20 | (50.0) |  |
| Co-morbidity/ Poor performance status | 2 | (6.3) | 3 | (7.5) |  |
| Local extent of disease | 5 | (15.6) | 7 | (17.5) |  |
| Patient choice | 8 | (25.0) | 10 | (25.0) |  |
| **T - Stage** |  |  |  |  |  |
| T1 | 1 | (3.1) | 0 | (0.0) |  |
| T2 | 3 | (9.4) | 10 | (25.0) |  |
| T3 | 21 | (65.6) | 25 | (62.5) |  |
| T4a | 5 | (15.6) | 3 | (7.5) |  |
| T4b | 2 | (6.3) | 2 | (5.0) |  |
| **N - Stage** |  |  |  |  |  |
| N0 | 15 | (46.9) | 16 | (40.0) |  |
| N1 | 13 | (40.6) | 17 | (42.5) |  |
| N2 | 4 | (12.5) | 6 | (15.0) |  |
| N3 | 0 | (0.0) | 1 | (2.5) |  |
| **Histologic grade** |  |  |  |  |  |
| G1 | 0 | (0.0) | 1 | (2.5) |  |
| G2 | 20 | (62.5) | 24 | (60.0) |  |
| G3 | 11 | (34.4) | 12 | (30.0) |  |
| Missing | 1 | (3.1) | 3 | (7.5) |  |
| **Screening TNM v7 Stage** |  |  |  |  |  |
| I | 1 | (3.1) | 1 | (2.5) |  |
| IIa | 3 | (9.4) | 6 | (15.0) |  |
| IIb | 10 | (31.3) | 14 | (35.0) |  |
| III | 18 | (56.3) | 19 | (47.5) |  |
| **Site of predominant tumour** |  |  |  |  |  |
| Upper 1/3 (14 to <24 cm) | 4 | (12.5) | 6 | (15.0) |  |
| Mid-point (24 to <32 cm) | 17 | (53.1) | 13 | (32.5) |  |
| Lower 1/3 (32-40 cm) | 11 | (34.4) | 21 | (52.5) |  |
| **Tumour type** |  |  |  |  |  |
| Squamous cell carcinoma | 27 | (84.4) | 31 | (77.5) |  |
| Adenocarcinoma/undifferentiated | 5 | (15.6) | 9 | (22.5) |  |
| **Median overall length of primary tumour (cm)**, IQR | 4.1, 3.0-5.7 | | 5.0, 3.0-6.0 | |  |
| **Total disease length (cm)**, IQR | 5.0, 3.1-6.8 | | 5.6, 3.6-7.0 | |  |
| **Baseline SUV_max_ – mean (SD)** | 12.9 (4.8) | | 17.2 (5.5) | |  |
| **% change in SUV_max_ to day 14 - median IQR*** | 16.3 (7.4-23.7) | | 45.7 (40.9-55.2) | |  |

*100 – (100 x (SUV_max_ at 14 days/SUV_max_ at baseline))

NB. n (%) unless otherwise specified

**Table S10. 24 Week Treatment Failure-Free Survival (TFFS) by PET response**

|  | **Squamous cell carcinoma** | | | | **Adenocarcinoma/undifferentiated** | | | |
| --- | --- | --- | --- | --- | --- | --- | --- | --- |
|  | **PET non-responders cisplatin+capecitabine** | | **PET responders** | | **PET non-responders cisplatin+capecitabine** | | **PET responders** | |
| **Patients enrolled** | 27 | | 31 | | 5 | | 9 | |
|  | n | % | n | % | n | % | n | % |
| Died/progressed prior to week 24 scan^A^ | 0 | 0.0 | 2 | 6.5 | 1 | 20.0 | 1 | 11.1 |
| Valid CT scan done^B^ | 27 | 100.0 | 26 | 83.9 | 4 | 80.0 | 8 | 88.9 |
| Progression outside RT volume | 2 | 7.4 | 1 | 3.2 | 0 | 0.0 | 0 | 0.0 |
| Valid endoscopy done ^C^ | 23 | 85.2 | 25 | 80.6 | 3 | 60.0 | 8 | 88.9 |
| Residual/persistent disease | 0 | 0.0 | 2 | 6.5 | 0 | 0.0 | 1 | 11.1 |
| Response assessable | 27 | 100.0 | 28^D^ | 90.3 | 5 | 100.0 | 9 | 100.0 |
| Failure-free | 25 | 92.6 | 22 | 78.6 | 4 | 80.0 | 7 | 77.8 |
| Failure | 2 | 7.4 | 6 | 21.4 | 1 | 20.0 | 2 | 22.2 |

^A^ Therefore no CT scan due at 24 weeks

^B^ Done at 24 weeks (+/-4 weeks) after start of treatment

^C^ Done at 24 weeks (+12/-4 weeks) after start of treatment

^D^ One patient had no residual disease on valid endoscopy but CT scan was not done. One patient had no residual disease on valid endoscopy but CT scan was done too early (although showed no progression). One patient withdrew from trial treatment and follow up due to chest pain and died at 9.5 months after randomisation due to metastatic oesophageal cancer.
